# Burden of long COVID among adults experiencing sheltered homelessness: a longitudinal cohort study in King County, WA between September 2020—April 2022

**DOI:** 10.1186/s12889-023-16026-7

**Published:** 2023-06-06

**Authors:** Sarah N. Cox, Emily M. Scott, Julia H. Rogers, Eric J. Chow, Jessica K. Wasse, Marco Carone, James P. Hughes, Helen Y. Chu

**Affiliations:** 1 Department of Epidemiology, University of Washington, WA Seattle, USA; 2 Department of Medicine, University of Washington, Seattle, USA; 3 Department of Medicine, University of Colorado, Denver, USA; 4 Public Health – Seattle & King County, Seattle, USA; 5 Fred Hutchinson Cancer Center, Vaccine and Infectious Disease Division, Seattle, USA; 6 Department of Biostatistics, University of Washington, Seattle, USA

**Keywords:** COVID-19, SARS-CoV-2, Homelessness, Long COVID, Post-COVID-19 conditions

## Abstract

**Background:**

People experiencing homelessness (PEH) are at increased risk for acquiring SARS-CoV-2, but the burden of long COVID in this population is unknown.

**Methods:**

We conducted a matched prospective cohort study to assess the prevalence, characteristics, and impact of long COVID among sheltered PEH in Seattle, WA between September 2020—April 2022. Adults ≥ 18 years, residing across nine homeless shelters with active respiratory virus surveillance, were eligible to complete in-person baseline surveys and interval follow-up phone surveys. We included a subset of 22 COVID-19-positive cases who tested positive or inconclusive for SARS-CoV-2 and 44 COVID-19-negative controls who tested negative for SARS-CoV-2, frequency matched on age and sex. Among controls, 22 were positive and 22 were negative for one of 27 other respiratory virus pathogens. To assess the impact of COVID-19 on the risk of symptom presence at follow-up (day 30–225 post-enrollment test), we performed log-linear regression with robust standard errors, adjusting for confounding by shelter site and demographic variables determined a priori.

**Results:**

Of 53 eligible COVID-19 cases, 22 (42%) completed ≥ 1 follow-up survey. While five (23%) cases reported ≥ 1 symptom at baseline, this increased to 77% (10/13) between day 30–59 and 33% (4/12) day 90 + . The most commonly reported symptoms day 30 + were fatigue (27%) and rhinorrhea (27%), with 8 (36%) reporting symptoms that interfered with or prevented daily activities. Four (33%) symptomatic cases reported receiving medical care outside of a medical provider at an isolation facility. Of 44 controls, 12 (27%) reported any symptoms day 90 + . Risk of any symptoms at follow-up was 5.4 times higher among COVID-19 cases compared to controls (95% CI: 2.7–10.5).

**Conclusions:**

Shelter residents reported a high prevalence of symptoms 30 + days after their SARS-CoV-2 detection, though few accessed medical care for persistent illness. The impact of COVID-19 extends beyond acute illness and may exacerbate existing challenges that marginalized populations face in maintaining their health and wellbeing.

**Supplementary Information:**

The online version contains supplementary material available at 10.1186/s12889-023-16026-7.

## Background

Severe acute respiratory syndrome coronavirus-2 (SARS-CoV-2) infection is associated with multiple prolonged symptoms and sequelae that can impact daily living, known as post-COVID-19 conditions or “long COVID” [1–4]. Symptoms of long COVID include fatigue, cough, difficulty breathing, loss of taste or smell, cognitive impairment, insomnia, and autonomic dysfunction [1–5]. The definition of long COVID continues to vary and evolve, highlighting our limited understanding of its nature and underlying mechanisms. However, the World Health Organization currently defines post-COVID-19 conditions as illness consisting of symptoms that are present three months after probable or confirmed SARS-CoV-2 infection, have a minimum duration of two months, and cannot be explained by an alternative diagnosis [2].

Studies have shown widely varying prevalence of long COVID during subsequent months following infection, higher in populations requiring hospitalization during acute COVID-19 illness [6] A meta-analysis of 54 studies, mostly of non-hospitalized individuals with symptomatic acute COVID-19 illness, estimated that 6.2% of individuals experienced ≥ 1 persistent symptom three months after acute illness [7] Long COVID’s clinical presentation, frequency, and impact may vary among different populations, [8] and there is a need to better understand prevalence and persistent outcomes among high-risk groups, particularly marginalized populations and those with barriers to accessing care.

The COVID-19 pandemic has disproportionately affected people experiencing homelessness (PEH), with congregate shelters often representing a hotspot for outbreaks [9–13]. To our knowledge, there are no currently published studies describing long COVID among PEH. We hypothesized that there would be a high burden of long COVID among PEH given elevated rates of underlying medical conditions and increased risk for severe COVID-19 disease compared to the general population [14, 15]. Moreover, surveys and interviews of patients with long COVID have reported significant, negative effects on physical and psychiatric health, employment, and access to medical care— impacts that may be amplified in unhoused populations and can exacerbate existing barriers to health, housing, and medical care [16–19].

This study aimed to characterize the burden and impact of long COVID among adult shelter residents in King County, Washington between September 2020—April 2022, using a prospective cohort of COVID-19-positive cases and COVID-19-negative controls, frequency matched on age and sex.

## Methods

### Study design

To assess long COVID among shelter residents, we used a frequency-matched prospective cohort study design, nested within the Seattle Flu Study’s (SFS) cross-sectional, community-based respiratory virus surveillance. As previously described, [20, 21] SFS instituted active routine surveillance three to six days per week among shelter residents and staff ≥ 3 months of age specifically for SARS-CoV-2 starting in March 2020.

### Study participants

Adults ≥ 18 years whose primary residence was at one of nine homeless shelters with active surveillance between 9/1/2020–5/31/2021 were eligible to participate in this sub-study (Table 1). This included a mix of adult, family, and young adult shelters, selected to be socio-demographically representative of King County’s sheltered PEH population (supplementary materials, Appendix 1, Table S1). Shelter sites were identified by the study team in collaboration with Public Health - Seattle & King County and community partners. Study enrollment was open to shelter residents and staff regardless of symptoms. Each shelter participant was limited to one enrollment and nasal swab per week unless they developed new or worsening cough or ≥ 2 acute respiratory illness symptoms (i.e., fever, cough, sore throat, dyspnea, rhinorrhea, myalgia, or headache) in the seven days following last enrollment.

**Table 1:** Participant characteristics by COVID-19 and Other Respiratory Virus (ORV) case status

	**COVID-19-positive cases (*n* = 22)**	**COVID-19-negative, ORV-positive controls (*n *= 22)**	**COVID-19-negative, ORV-negative controls (*n* = 22)**	**Overall (*N* = 66)**
**Age (years)**^**†**^	45.0 [20.0, 66.0]	37.5 [18.0, 72.0]	44.5 [21.0, 64.0]	44.0 [18.0, 72.0]
**Sex (biological)**				
Male	11 (50.0%)	11 (50.0%)	11 (50.0%)	33 (50.0%)
Female	10 (45.5%)	10 (45.5%)	10 (45.5%)	30 (45.5%)
Prefer not to say	1 (4.5%)	1 (4.5%)	1 (4.5%)	3 (4.5%)
**Hispanic ethnicity**				
No	19 (86.4%)	19 (86.4%)	16 (72.7%)	54 (81.8%)
Yes	2 (9.1%)	2 (9.1%)	5 (22.7%)	9 (13.6%)
Prefer not to say	1 (4.5%)	1 (4.5%)	1 (4.5%)	3 (4.5%)
**Race**				
American Indian or Alaska Native	1 (4.5%)	1 (4.5%)	2 (9.1%)	4 (6.1%)
Asian	0 (0.0%)	1 (4.5%)	2 (9.1%)	3 (4.5%)
Black or African American	10 (45.5%)	9 (40.9%)	7 (31.8%)	26 (39.4%)
Native Hawaiian or other Pacific Islander	1 (4.5%)	1 (4.5%)	2 (9.1%)	4 (6.1%)
White	4 (18.2%)	4 (18.2%)	6 (27.3%)	14 (21.2%)
Multiracial	2 (9.1%)	2 (9.1%)	0 (0.0%)	4 (6.1%)
Other	2 (9.1%)	0 (0.0%)	0 (0.0%)	2 (3.0%)
Prefer not to say	2 (9.1%)	4 (18.2%)	3 (13.6%)	9 (13.6%)
**Language**				
English	19 (86.4%)	22 (100.0%)	21 (95.5%)	62 (93.9%)
Spanish	2 (9.1%)	0 (0.0%)	1 (4.5%)	3 (4.5%)
Tigrinya	1 (4.5%)	0 (0.0%)	0 (0.0%)	1 (1.5%)
**Education**				
Less than high school graduate	4 (18.2%)	2 (9.1%)	2 (9.1%)	8 (12.1%)
Graduated high school/obtained GED	7 (31.8%)	6 (27.3%)	6 (27.3%)	19 (28.8%)
Some college^‡^	3 (13.6%)	6 (27.3%)	8 (36.4%)	17 (25.8%)
Bachelor's or advanced degree	1 (4.5%)	7 (31.8%)	6 (27.2%)	14 (21.2%)
Prefer not to say	1 (4.5%)	1 (4.5%)	0 (0.0%)	2 (3.0%)
Missing	6 (27.3%)	0 (0.0%)	0 (0.0%)	6 (9.1%)
**Employed**				
No	13 (59.1%)	14 (63.6%)	9 (40.9%)	36 (54.5%)
Yes	3 (13.6%)	8 (36.4%)	13 (59.1%)	24 (36.4%)
Missing	6 (27.3%)	0 (0.0%)	0 (0.0%)	6 (9.1%)
**Income**				
≤ $25,000	10 (45.5%)	10 (45.5%)	15 (68.2%)	35 (53.0%)
> $25,000	1 (4.5%)	6 (27.3%)	5 (22.7%)	12 (18.2%)
Don't know or prefer not to say	5 (22.7%)	6 (27.3%)	2 (9.1%)	13 (19.7%)
Missing	6 (27.3%)	0 (0.0%)	0 (0.0%)	6 (9.1%)
**Insurance**				
Private	0 (0.0%)	6 (27.3%)	5 (22.7%)	11 (16.7%)
Government	15 (68.2%)	12 (54.5%)	12 (54.5%)	39 (59.1%)
None	0 (0.0%)	3 (13.6%)	4 (18.2%)	7 (10.6%)
Prefer not to say	1 (4.5%)	1 (4.5%)	1 (4.5%)	3 (4.5%)
Missing	6 (27.3%)	0 (0.0%)	0 (0.0%)	6 (9.1%)
**Duration of homelessness**				
6 months or less	2 (9.1%)	5 (22.7%)	5 (22.7%)	12 (18.2%)
7–12 months	1 (4.5%)	3 (13.6%)	4 (18.2%)	8 (12.1%)
13–24 months	4 (18.2%)	3 (13.6%)	0 (0.0%)	7 (10.6%)
Over 24 months (2 years)	8 (36.4%)	4 (18.2%)	5 (22.7%)	17 (25.8%)
Prefer Not to Say	1 (4.5%)	1 (4.5%)	1 (4.5%)	3 (4.5%)
Missing	6 (27.3%)	6 (27.3%)	7 (31.8%)	19 (28.8%)
**Shelter Type**				
Mixed gender, ≥ 18 years	11 (50.0%)	9 (40.9%)	7 (31.8%)	27 (40.9%)
Mixed gender, 18—25 years	0 (0.0%)	2 (9.1%)	1 (4.5%)	3 (4.5%)
Female, ≥ 18 years	0 (0.0%)	2 (9.1%)	2 (9.1%)	4 (6.1%)
Male, ≥ 18 years	2 (9.1%)	2 (9.1%)	2 (9.1%)	6 (9.1%)
Male, ≥ 50 years	1 (4.5%)	0 (0.0%)	3 (13.6%)	4 (6.1%)
Mixed gender, all ages	8 (36.4%)	7 (31.8%)	7 (31.8%)	22 (33.3%)
**Any comorbidities**^**§**^	6 (27.3%)	7 (31.8%)	6 (27.3%)	19 (28.8%)
**Smoking status**				
None	12 (54.5%)	11 (50.0%)	15 (68.2%)	38 (57.6%)
Tobacco products	9 (40.9%)	10 (45.5%)	6 (27.3%)	25 (37.9%)
Prefer not to say	1 (4.5%)	1 (4.5%)	1 (4.5%)	3 (4.5%)
**Any symptoms at enrollment**	5 (22.7%)	2 (9.1%)	4 (18.2%)	11 (16.7%)
**Follow-up time since enrollment (days)**^**†**^	72.0 [35.0, 183.0]	180 [96.0, 223.0]	123 [96.0, 188.0]	126 [35.0, 223.0]
**Follow-up season**^**‖**^				
Fall	1 (4.55%)	16 (72.7%)	13 (59.1%)	30 (45.5%)
Spring	3 (13.6%)	0 (0.0%)	0 (0.0%)	3 (4.5%)
Winter	18 (81.8%)	0 (0.0%)	0 (0.0%)	18 (27.3%)
Summer	0 (0.0%)	6 (27.3%)	9 (40.9%)	15 (22.7%)
**Any symptoms at follow-up**^**a**^	9 (40.9%)	5 (22.7%)	7 (31.8%)	21 (31.8%)

^†^ Median [Min, Max]

^‡^ Some college includes: vocational training, associate's degree

^**§**^ Any comorbidities includes: asthma, blood disorders, cancer, chronic obstructive pulmonary disease or emphysema, immunosuppression, liver disease, heart disease, diabetes, neurologic conditions, or aspirin therapy

^**‖**^ Seasons defined by astronomical season in the Northern Hemisphere (using equinox and solstice dates)

^a^ Any symptoms at follow-up represents a single follow-up time point between day 30–225 post-enrollment. If a COVID-19 case had more than one follow-up survey complete between day 30–225 post enrollment, the median time point was selected and used

COVID-19 cases tested positive or inconclusive for SARS-CoV-2 and agreed to participate in follow-up survey(s). Each case was offered an isolation unit within the shelter or an off-site isolation unit by Public Health - Seattle & King County where they had access to medical care. Controls were those who agreed to participate and were: (1) negative for SARS-CoV-2 and other respiratory viruses (ORVs) (COVID-19-negative, ORV-negative controls, *n* = 22), or (2) negative for SARS-CoV-2 but positive for an ORV (COVID-19-negative, ORV-positive controls, *n* = 22) (supplementary materials, Appendix 1, Figure S1). All controls were asked about any infection at follow-up. Participants who self-reported testing positive or inconclusive for SARS-CoV-2 before their follow-up interview were excluded from the control group. Cases and controls were frequency-matched by sex and age.

### Data collection

Self-collected mid-nasal swabs and enrollment surveys were provided by all participants. Samples were tested for 27 respiratory pathogens using a TaqMan reverse transcriptase polymerase chain reaction (PCR) [21]. The study team conducted interval phone follow-up surveys between day 5–365 after initial swabbing. COVID-19 cases had follow-up attempts at approximately 5, 10, 30, 60, 180, and 365 days post-enrollment. COVID-19 controls had one follow-up between 90–225 days post-enrollment. Control surveys did not state the SARS-CoV-2 or other PCR test results and instead, only referenced the date at which testing was completed.

Study staff called participants on the phone number provided during baseline enrollment when available; if not, study staff coordinated with shelter management to connect with participants’ rooms if still residing at the shelter site. For each individual, three contact attempts were made at each time point. For all participants who primarily spoke a language other than English, a certified medical interpreter was used to complete the survey. All survey data were collected and entered electronically in Research Electronic Data Capture (REDCap). The enrollment questionnaire (Appendix 2) [21] and long COVID follow-up questionnaires (Appendix 3 and 4) are included in supplementary materials. We offered $10 gift cards to compensate COVID-19-positive participants for their time at 180 and 365 day survey time points. This study was approved by the Human Subjects Division of the University of Washington Institutional Review Board (STUDY00007800).

### Main measures

The primary exposure was COVID-19 case status (binary), where COVID-19-positive cases (who tested positive or inconclusive for SARS-CoV-2) represent the “exposed” group and COVID-19-negative controls (who tested negative for SARS-CoV-2) represent the “unexposed” group. The primary outcome was one or more symptom(s) at follow-up between day 30–225 post-enrollment test (binary). For COVID-19 cases with more than one follow-up survey completed between day 30–225 post-enrollment, the survey from the median time point was selected and used. Symptoms included subjective fever, headache, cough, chills, sweats, sore throat, rhinorrhea, fatigue, myalgias, trouble breathing, ear pain or discharge, nausea or vomiting, rash, and loss of smell or taste. The enrollment questionnaire included data on underlying medical conditions, smoking status, shelter use, and duration of homelessness. Underlying medical conditions included asthma, blood disorders (e.g., sickle cell disease), cancer, chronic obstructive pulmonary disease or emphysema, chronic bronchitis, immunosuppression, liver disease, heart disease, or diabetes. Follow-up questionnaires collected information on residual symptoms, receipt of medical care, impact on work or school absenteeism, impact on daily activities, and the CDC Healthy Days Core Module and Activities Limitations Module to assess health-related quality of life (HR-QOL) at each time point [22]. If a participant did not complete the day five questionnaire, they were asked to recall their HR-QOL prior to COVID-19 diagnosis on the first follow-up questionnaire.

### Data analysis

Descriptive statistics were used to characterize our primary measures and covariates of interest at various survey time points. We performed log-linear regression to assess the association between exposure and risk of presence of symptom(s) at follow-up between day 30–225. Specifically, Poisson regression models were fitted using generalized estimating equations (GEE) to account for possible correlation within shelter sites. Risk ratio (RR) estimates were obtained, and Wald-based confidence intervals and hypothesis tests were conducted using robust standard error estimates. The primary analysis examined cases versus controls, while the secondary analysis used a three-level case status exposure: (1) COVID-19-positive case, (2) COVID-19-negative and ORV-negative control, and (3) COVID-19-negative and ORV-positive control. In both models, we assessed for interaction between case status and time since enrollment, as we hypothesized that symptoms reported among controls would remain constant while symptoms among cases would decline over time. While controls were not at risk for long COVID, it was important to assess symptoms reported in a comparator group given higher rates of chronic, underlying medical conditions among PEH.

Key confounders identified a priori from the literature included follow-up time since enrollment, [23] follow-up season, [24] race, [25–28] any comorbidities, [1, 4, 25, 29–35] income, [29] and smoking status [1, 4, 25, 29–35]. Other covariates deemed potential confounders included duration of homelessness, [13, 36–41] employment, insurance, [25, 42] education, [42, 43] and Hispanic ethnicity [25, 27, 33, 43]. To more flexibly model the effect of follow-up time since enrollment (centered at 90 days) on the log RR of symptom(s) at follow-up, we used restricted cubic splines with three knots (at the fifth smallest, median, and fifth largest data points) [44, 45]. In sensitivity analyses, both primary and secondary analyses adjusted for all confounders identified a priori using a bivariate screening procedure to reduce the number of covariates included, (≥ 5%-point difference across strata between both the frequency of the exposure by covariate level and the frequency of the outcome by covariate level), followed by a forward selection procedure (starting with strongest confounders in our data, ensuring that the estimate of interest (i.e., log RR) did not have an absolute change of more than 0.5 and that inclusion/exclusion of the null in the confidence interval did not change). We also present results of the full model. All analyses were performed using R Statistical Software Version 4.0.3.

## Results

### Participant characteristics: COVID-19-positive cases

Between 9/1/2020–5/31/2021, 53 adult shelter residents tested positive (*n* = 50) or inconclusive (*n* = 3) for SARS-CoV-2 (supplementary materials, Appendix 1, Figure S1). Of these cases, 22 (42%) completed one or more follow-up questionnaires and were included in this analysis (Table 1; supplementary materials, Appendix 1, Table S2). Cases reached were similar to those unable to be reached with the exception of providing a phone number to contact (77% vs. 48%) (supplementary materials, Appendix 1, Table S3). The median age of cases was 45 years (range: 20, 66). Half (*n* = 11) reported male sex at birth and most identified as either Black/African American (46%, *n* = 10) or White (18%, *n* = 4). The majority of cases (55%, *n* = 12) reported chronic homelessness (duration of ≥ 1 year), having health insurance (68%) and being unemployed (59%). Six (27%) self-reported ≥ 1 underlying medical condition and nine (41%) indicated that they were smokers. Three (8%) of COVID-19-positive cases were co-infected with an ORV at enrollment, all of which were rhinovirus.

#### Self-reported symptoms and medical care

**Fig. 1:**
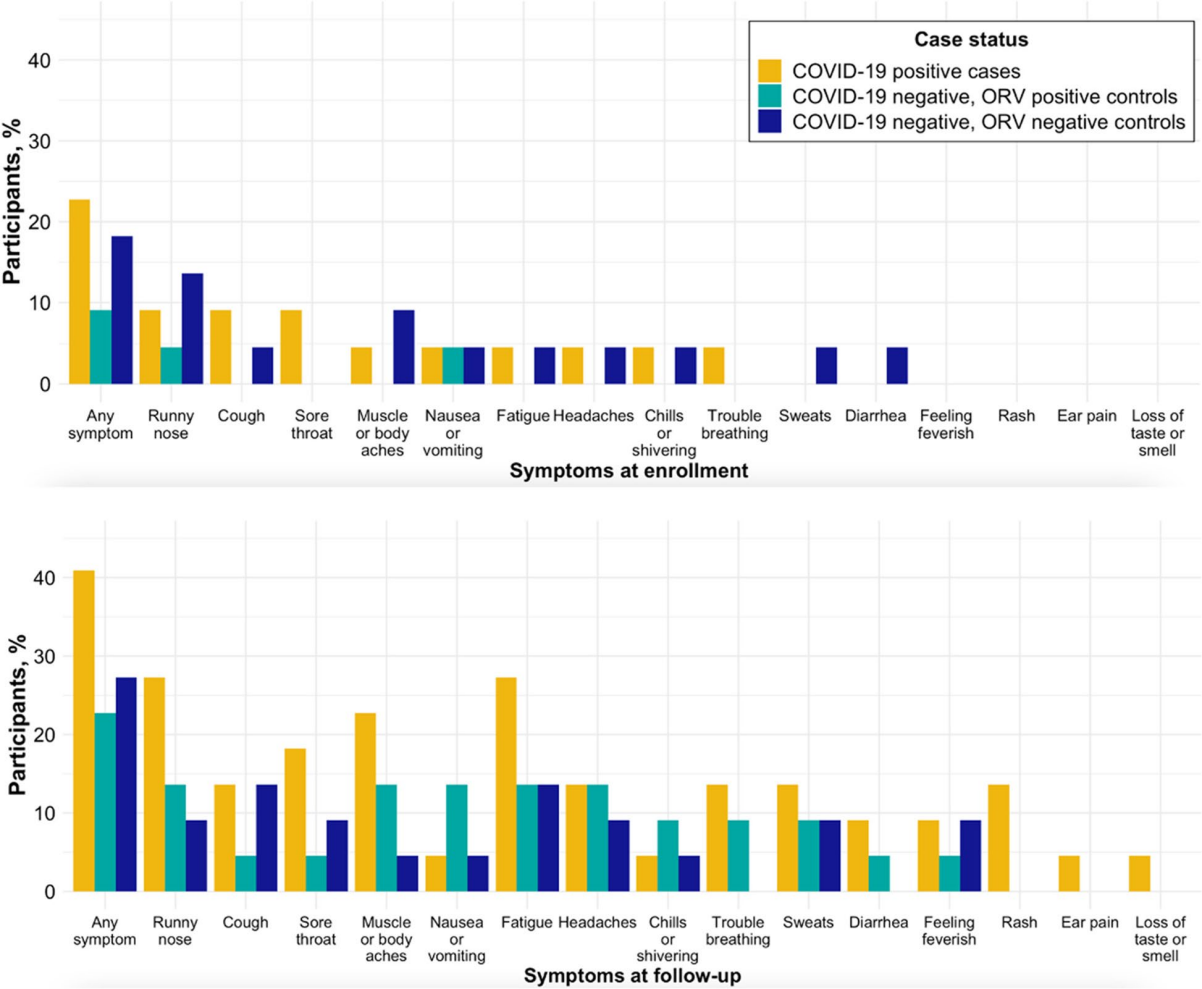
Symptoms reported by COVID-19 & Other Respiratory Virus (ORV) case status day 30–225 since enrollment

**Fig. 2:**
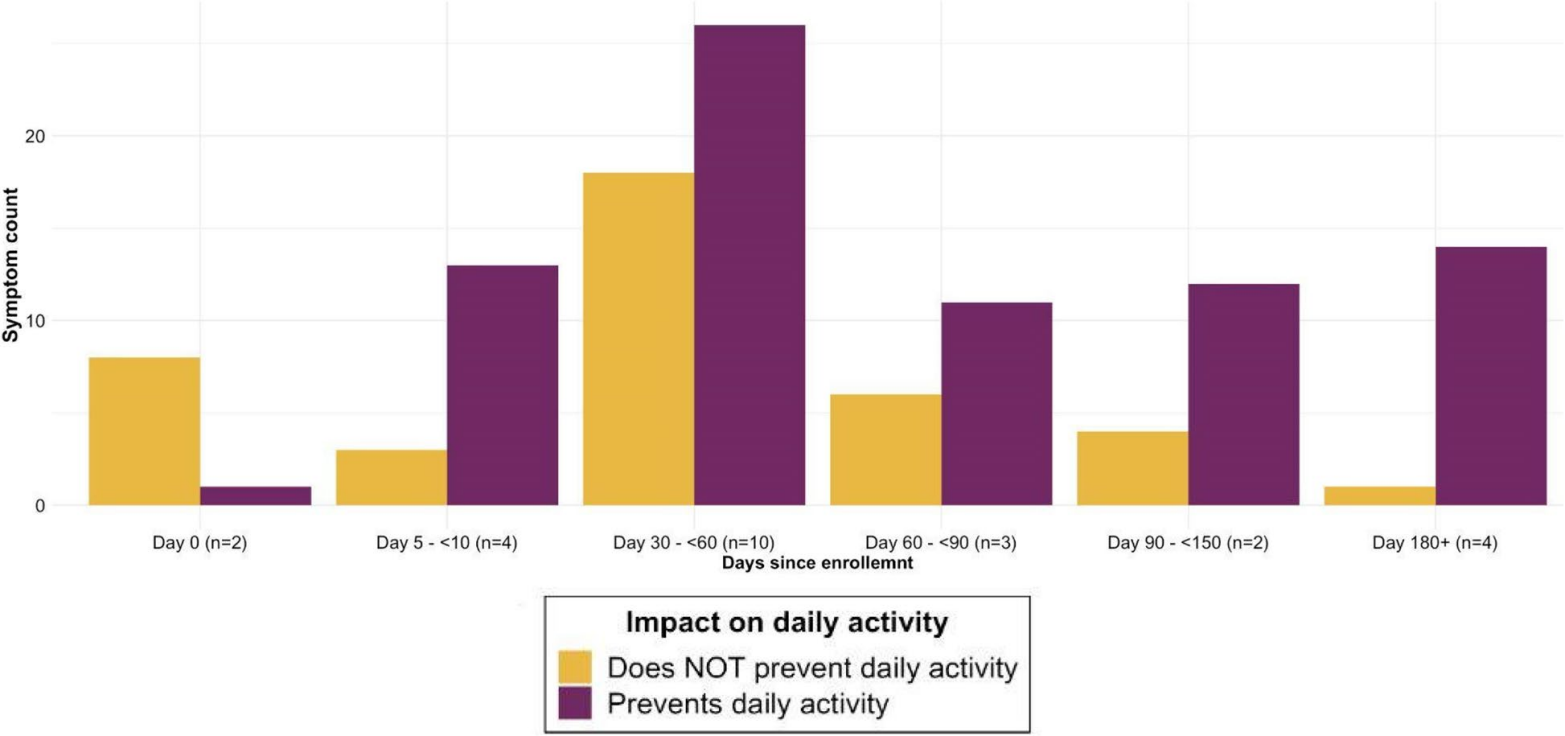
Symptom count and impact among symptomatic COVID-19 cases at follow-up time points day 30–225 since enrollment

Five (23%) cases at baseline enrollment were symptomatic, of which four indicated having one symptom, while one participant indicated having eight symptoms (Fig. 1; supplementary materials, Appendix 1, Figure S2). The mean time to follow-up after COVID-19 diagnosis was 85 ± 40 days (Table 1; supplementary materials, Appendix 1, Figure S3). The most common symptoms reported at follow-up were fatigue (27%), runny nose (27%), muscle or body aches (23%), and sore throat (18%) (Fig. 1). All four cases reached for follow-up between day 5- < 10 were symptomatic. Approximately 77% of COVID-19 cases (10/13) surveyed had symptoms between day 30- < 60, with an even distribution of symptoms preventing, interfering with, and not impacting daily activities. The prevalence of persistent symptoms after acute COVID-19 decreased to 30% at day 60- < 90, 33% at day 90- < 180, and 33% at day 180 + . By day 60 + , 43% of people who reported symptoms indicated that at least one prevented daily activity (Fig. 2).

**Fig. 3:**
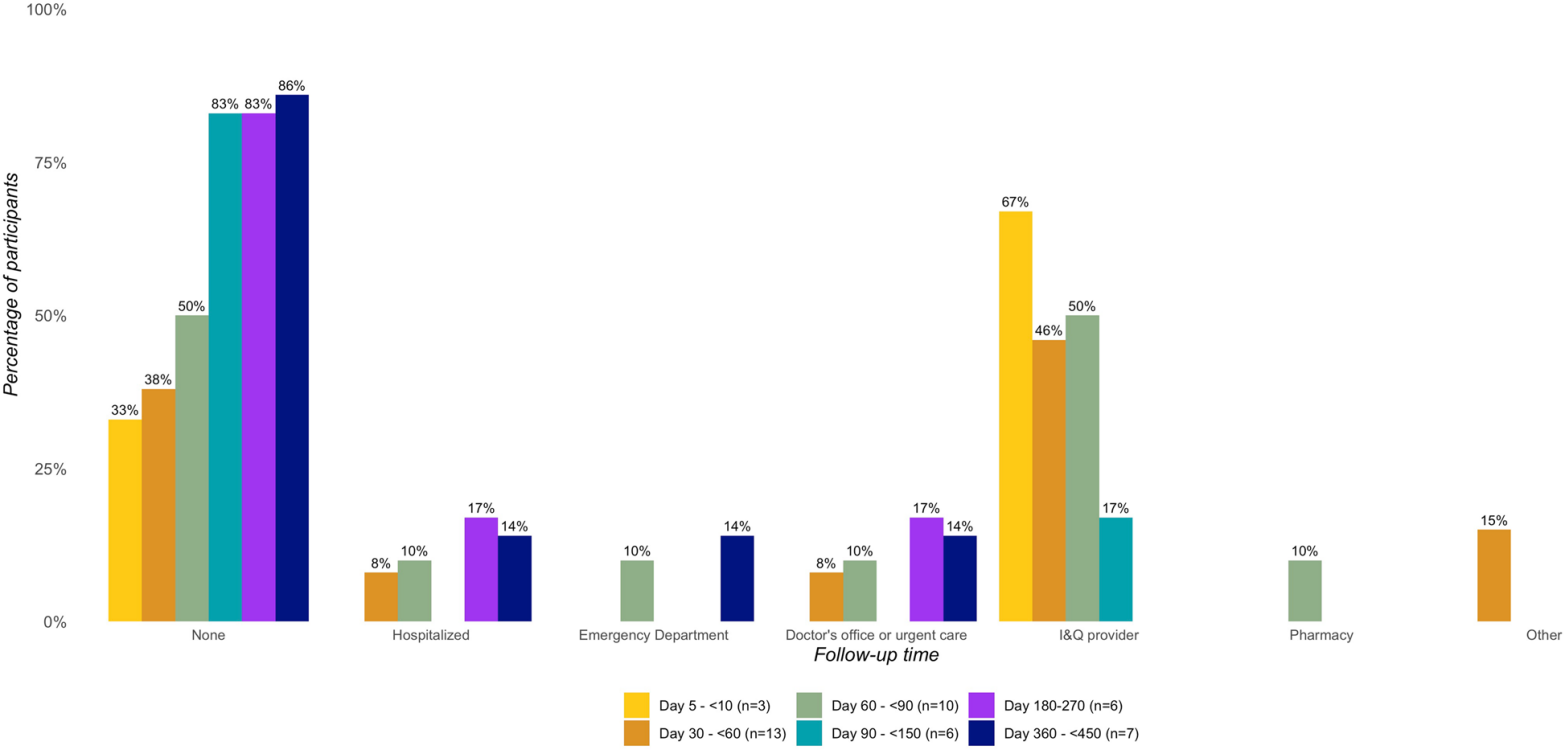
Medical care received in the last three months since positive/inconclusive SARS-CoV-2 test by time since enrollment

The majority of COVID-19-positive cases (70%, *n* = 18) reported not receiving medical care outside of a provider at an isolation and quarantine facility day 30 + (Fig. 3). The proportion of COVID-19-positive cases without medical care in the last three months increased over time. While approximately 35% received no medical care ≤ 60 days since SARS-CoV-2 infection, this increased to approximately 85% without medical care 180 + days since infection. Only four participants sought care elsewhere.

#### Self-reported HR-QOL and impact on activities of daily living

**Fig. 4:**
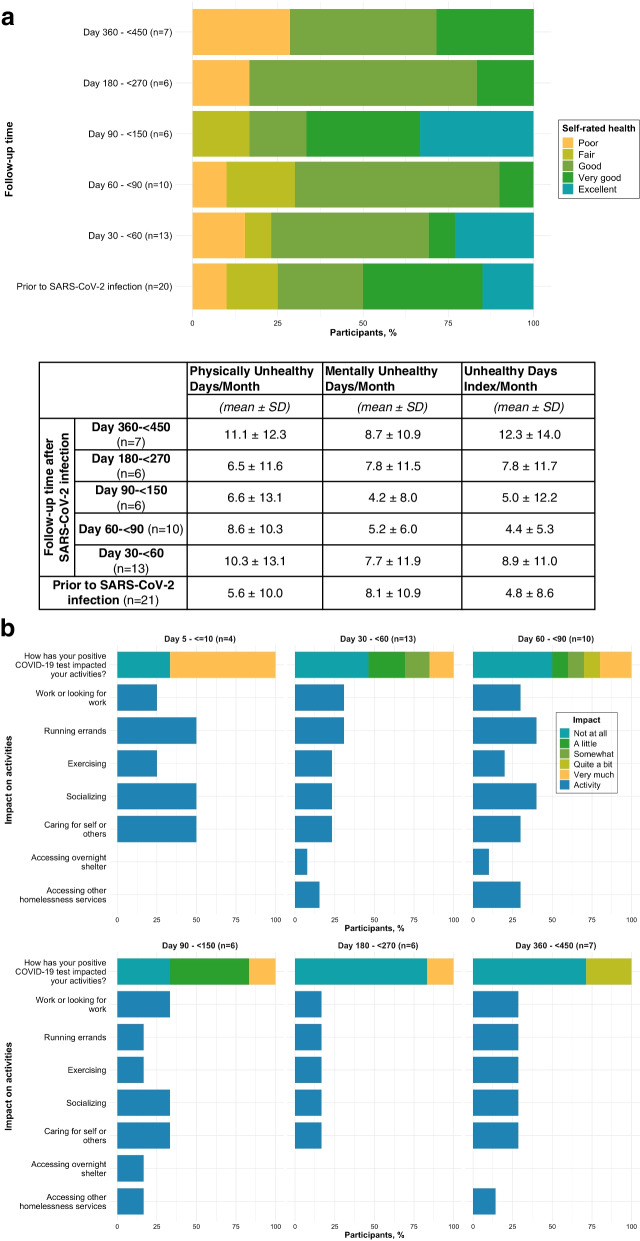
**a** Health-related quality of life: self-reported health and CDC Healthy Days. **b** Impact of positive/inconclusive SARS-CoV-2 test on daily activities

Participants' self-rated health prior to COVID-19 illness and at follow-up intervals is shown in Fig. 4a. The mean number of days that physical health was reported to be "NOT good" and that poor physical or mental health prevented usual activities in the past 30 days increased for all but one follow-up time point compared to prior to SARS-CoV-2 infection. The mean number of days that mental health was reported to be "NOT good" in the past 30 days initially decreased then returned to baseline compared to prior to SARS-CoV-2 infection. In approximately half of cases, a positive COVID-19 test impacted daily activities between day 30- < 150 (Fig. 4b). The most commonly impacted activities included work or looking for work (30%-33%), socializing (23%-40%), running errands (17%-40%), and caring for self or others (23%-33%).

### Participant characteristics: COVID-19-negative controls

Among controls (*n* = 44), a smaller proportion reported chronic homelessness (27%), unemployment (52%), or that they were smokers (36%), while a larger proportion had educational attainment higher than high school (61%) or were insured (80%) compared to COVID-19 cases (Table 1; supplementary materials, Appendix 1, Table S4). COVID-19-negative controls reported lower overall prevalence of symptoms at baseline (13% vs. 23%) and follow-up (27% vs. 41%) compared to COVID-19-positive cases, despite increasing in both groups.

Among COVID-19-negative ORV-positive controls, 73% (*n* = 16) tested positive for rhinovirus. ORVs identified included seasonal coronavirus (*n* = 3), parainfluenza (*n* = 1), metapneumovirus (*n* = 1), and adenovirus (*n* = 1). COVID-19-negative controls who were ORV-positive (compared to ORV-negative) had a lower prevalence of symptoms at both baseline (9% vs. 18%) and follow-up (23% vs. 27%). The mean time to follow-up after COVID-19 diagnosis was 151 ± 40 days versus controls ORV-positive (169 ± 39) and ORV-negative (132 ± 31).

### Risk of persistent symptoms

Table 2 presents risk of persistent symptoms and sensitivity analyses from regression models. In the primary analysis model adjusting only for the confounders of follow-up season and time (in addition to age and sex due to frequency matching in study design), the estimated risk of symptoms at follow-up was 5.4 times higher among COVID-19 cases compared to controls (95% CI: 2.7–10.5). This relationship remained significant in sensitivity analyses when adjusting for additional confounders (RR = 5.7, 95% CI: 1.1–30.3) and in the full model including all potential confounders (Table 2). In the secondary analysis separating COVID-19-negative controls by ORV result, the estimated risk of symptoms at follow-up were 6.3 (95% CI: 1.8–21.7) and 4.2 (95% CI: 1.5–11.9) times higher among COVID-19 cases compared to ORV-positive and negative controls, respectively. After adjusting for the same set of additional confounders, this relationship remained significant among COVID-19-negative, ORV-positive controls (RR = 7.1, 95% CI: 1.2–41.7). However, among COVID-19-negative, ORV-negative controls the estimated risk was no longer statistically significant after adjustment. We found no evidence of interaction between case status and time since enrollment in either model, and thus presented models do not include an interaction term.

**Table 2:** Log-linear regression models to assess risk of symptom(s) at follow-up

	**aRR**	**95% CI**	***p*-value**
***Primary analysis***^†^			
** Model A: No covariates**^a^			
Case (vs. control)	1.500	(0.949—2.371)	0.083
** Model B: Add covariates time and follow-up season only**^b^			
Case (vs. control)	5.365	(2.731—10.539)	< 0.001*
** Model C: Sensitivity analysis—expanded model**^c^			
Case (vs. control)	5.735	(1.086—30.285)	0.040*
** Model D: Sensitivity analysis—full model**^d^			
Case (vs. control)	8.902	(2.015—39.324)	0.004*
***Secondary analysis***^‡^			
** Model A: No covariates**^a^			
Case (vs. Control, ORV-positive)	1.799	(0.663—4.902)	0.249
Case (vs. Control, ORV-negative)	1.285	(0.791—2.092)	0.311
** Model B: Add covariates time and follow-up season only**^b^			
Case (vs. Control, ORV-positive)	6.250	(1.805—21.739)	0.004*
Case (vs. Control, ORV-negative)	4.237	(1.499—11.905)	0.006*
** Model C: Sensitivity analysis—expanded model**^c^			
Case (vs. Control, ORV-positive)	7.143	(1.206—41.667)	0.030*
Case (vs. Control, ORV-negative)	4.237	(0.597—30.303)	0.149
** Model D: Sensitivity analysis—full model**^d^			
Case (vs. Control, ORV-positive)	11.494	(2.045—62.500)	0.006*
Case (vs. Control, ORV-negative)	6.494	(1.292—32.258)	0.023*

^†^ Primary analysis uses binary exposure (*COVID-19-positive case* vs. *COVID-19-negative control*)

^‡^ Secondary analysis uses categorical exposure (*COVID-19-positive case* vs. *COVID-19-negative control, ORV*-positive vs. *COVID-19 control, ORV-negative*)

^a^ Model A represents the model with no covariates, adjusted only for age and sex due to frequency matching in study design

^b^ Model B builds upon Model A by also including adjustment for time since enrollment via 4 restricted cubic splines (centered at day 90) and follow-up season (categorical: fall, spring, summer, winter). As we found no evidence of interaction between case status and time since enrollment in either model, the interaction term was not included in the presented results

^c^ Model C builds upon Model B by also including adjustment for key covariates identified a priori, including race (categorical: White, Black/African American, Other), any comorbidities (binary: yes, no), income (binary: ≥ $25,000, < $25,000) and smoking status (binary: smoker, non-smoker), and additional covariates added during bivariate and forward selection screening procedures, including duration of homelessness (categorical: < 12 months, 12 + months, prefer not to say/missing) and employment (binary: yes, no)

^d^ Model D builds upon Model C by also including adjustment for all remaining potential covariates, including insurance (binary: yes, no), education (binary: high school or less, some college or more), and hispanic ethnicity (binary: yes, no)

^*^p-value < 0.05

## Discussion

Our study is the first to characterize long COVID specifically in PEH, a population that has been demonstrated to be at increased risk for acute COVID-19 illness and faces multiple barriers to accessing medical care and participating in longitudinal research studies. We found that among PEH in the Seattle metropolitan region, COVID-19-positive cases were at five times higher risk of persistent symptoms between 30–225 days post-testing when compared to COVID-19-negative controls. The prevalence of persistent symptoms after SARS-CoV-2 infection decreased with time. Approximately 30% of those with persistent symptoms reported ≥ 1 that prevented daily activity at all follow-up time points, yet the majority did not seek medical care despite the substantial burden and impact of their symptoms.

The prevalence of persistent COVID-19 symptoms in our sample of sheltered PEH between day 30–225 appears to be higher than the estimated 10–35% cited in a meta-analysis of patients in the general population of high-income countries after mild SARS-CoV-2 infection [46]. However, given our small sample of cases who tested positive or inconclusive for SARS-CoV-2, results should be interpreted with caution. Additionally, we caution against drawing excessive conclusions from our findings, especially beyond descriptive statistics for COVID-19 cases within each follow-up interval presented in Figs. 2, 3 and 4. In a longitudinal prospective cohort from Washington state, approximately 33% of outpatients reported at least one persistent symptom at six months post-infection [47]. These estimates are much higher than the estimated 6% global prevalence of at least one persistent fatigue, respiratory, or cognitive symptom three months after symptomatic acute COVID-19 illness [7]. The most common long COVID symptoms associated with SARS-CoV-2 infection in our study were similar to those recognized in previous studies, such as fatigue [46] and respiratory symptoms [7]. A surprising finding was that 27% of COVID-19-negative controls reported symptoms at follow-up between day 30–225, with a higher prevalence among ORV-negative vs. ORV-positive controls. While the frequency of chronic symptoms similar to post-COVID-19 conditions may explain our higher prevalence of long COVID compared to other studies, adjusting for underlying conditions we estimated that PEH with a positive SARS-CoV-2 test at enrollment were at over five times increased risk of persistent COVID-19 symptoms compared to those with a negative test. This demonstrates the impact of COVID-19 illness on PEH beyond the increased risk of acute infection.

Our study suggests that long COVID may exacerbate existing challenges that PEH face in health and wellbeing [48–50]. Persistent COVID-19 symptoms impacted participants’ key activities such as employment, ability to care for themselves and others, and access to homelessness services. Participants also reported worsened HR-QOL from time of positive tests to follow-up. Despite the high prevalence of impactful persistent COVID-19 symptoms and worse overall health, few of our participants sought medical care related to their symptoms. PEH face multiple barriers in healthcare access that result in lower health-seeking behavior or inability to seek care [51, 52]. Thus, not only does the addition of persistent COVID-19 symptoms to existing survival demands and chronic illness burden experienced by PEH have the potential to worsen health and socioeconomic disparities between unhoused and housed populations, but PEH are less likely to receive care to mitigate the impact of long COVID.

### Strengths and limitations

Our study has several strengths and limitations. This is the first study to describe the impact of long COVID among PEH, a population that experiences a disproportionate burden of acute COVID-19 illness but faces barriers to participating in longitudinal research studies due to housing instability, daily survival demands, and variable access to phones and other communication methods. Most studies on long COVID have focused on advantaged populations in high- and middle-income countries. Thus, additional research focusing on vulnerable and neglected communities, including PEH, is needed to address health inequities [53]. We were able to follow some participants up to one year following their positive or inconclusive SARS-CoV-2 test and evaluate the impact of long COVID on daily activities and quality of life.

Our study’s conclusions are limited by the small sample size, inconsistent follow-up, and differential distribution of follow-up time between cases and controls. Over half of COVID-19 cases and controls were lost to follow-up and unreachable after enrollment. This may lead to differential misclassification of the outcome, where ascertainment of symptoms at follow-up is influenced by COVID-19 case status (e.g., cases may experience worse outcomes and thus be harder to reach, or experience better outcomes and thus not be interested in participating), which could falsely exaggerate or minimize the true association by biasing the observed results upward or downward, but not predictably so. As differential follow-up time between cases and controls is a strong confounder, we chose to include time since enrollment within our regression models using restricted cubic splines to improve upon the flexibility of our model with relative parsimony [54]. However, there still may be residual confounding from differential follow-up that was unaccounted for in our models. While our model adjusted for follow-up time and season, to avoid overfitting and ensure its face validity we did not include all confounders in the primary model [54]. However, sensitivity analyses including key confounders and the full model with all measured potential cofounders resulted in similar conclusions.

Unlike many studies, [1, 53] we included a control group of PEH who tested negative for SARS-CoV-2, including half who tested positive for an ORV. However, we did not collect data on self-reported re-infection for cases. As enrollment in weekly surveillance was optional, it is possible that controls had COVID-19 that was never identified and thus not self-reported. Moreover, the fact that symptoms were self-reported may also result in differential misclassification of the outcome, specifically regarding participants’ ability to distinguish between long COVID-related symptoms and symptoms related to other chronic diseases. Again, this type of misclassification leads to a less predictable direction of the bias on the observed risk ratio. Lastly, we only included sheltered PEH and did not capture the experience of long COVID in unsheltered PEH.

## Conclusions

Shelter residents reported a high prevalence of persistent symptoms 30 + days after their SARS-CoV-2 detection, with a significantly higher risk of symptoms at follow-up among COVID-19 cases compared to COVID-19-negative controls. Symptoms impacted participants’ ability to perform key daily activities, yet few participants accessed medical care for persistent illness. The impact of COVID-19 extends beyond acute illness and long COVID may exacerbate existing challenges that PEH face in maintaining their health and wellbeing. These findings provide a preliminary understanding of long COVID in the homeless community to inform public health measures and health service resource allocation among PEH.

## Supplementary Information

**Additional file 1: Appendix 1. **Supplemental Tables and Figures.

**Additional file 2: Appendix 2. **Enrollment Questionnaire.

**Additional file 3: Appendix 3. **COVID-19 Case Follow-up Questionnaire.

**Additional file 4. Appendix 4. **COVID-19 Control Follow-up Questionnaire.

## Data Availability

The data presented in this study are available on request from the corresponding author.

## Abbreviations

GEE: Generalized Estimating Equations
HR-QOL: Health-Related Quality of Life
ORVs: Other Respiratory Viruses
PEH: People Experiencing Homelessness
PCR: Polymerase Chain Reaction
REDCap: Research Electronic Data Capture
RR: Risk Ratio
SFS: Seattle Flu Study
SARS-CoV-2: Severe Acute Respiratory Syndrome Coronavirus-2
