# Supplementary material for: Burden of long COVID among adults experiencing sheltered homelessness: a longitudinal cohort study in King County, WA between September 2020—April 2022
Source: BMC Public Health. 2023 Jun 6;23:1079. doi: 10.1186/s12889-023-16026-7 (PMC10241609; doi:10.1186/s12889-023-16026-7)
Supplement: Supplementary file 1 — Additional file 1: Appendix 1. Supplemental Tables and Figures. [file 12889_2023_16026_MOESM1_ESM.docx]

**Table S1. SFS Shelter sites where sample collection occurred, 1 October 2019 – 31 May 2021**

| **Shelter** | **Maximum capacity** | **Resident sex** | **Resident age range** | **Sleeping arrangements available** |
| --- | --- | --- | --- | --- |
| A | 60 | Female | ≥ 18 years | Communal bunk beds |
| B | 100 | Mixed | ≥ 18 years | Communal bunk beds |
| C | 45 | Mixed | 18 - 25 years | Communal floor mats and bunks beds |
| D | 185 | Mixed | All ages ^+^ | Private rooms / shared rooms / communal floor mats |
| E | 70 | Mixed | All ages ^+^ | Private rooms / shared rooms / communal floor mats |
| F | 60 | Male | ≥ 18 years | Communal bunk beds |
| G | 275 | Mixed | ≥ 18 years | Private rooms / shared rooms |
| H | 275 | Mixed | All ages ^+^ | Private rooms / shared rooms |
| I | 45 | Male | ≥ 50 years | 5 person dorms |
| J* | 34 | Male | ≥ 18 years | Individual open cubicles |
| K** | 75 | Mixed | ≥ 18 years | Individual open cubicles |

^+^All ages= family shelter

^*^*Opened / data collection began 3 December 2020 to replace Shelter F*

*^**^ Opened / data collection began 3 December 2020 to replace Shelter B*

**Table S2. Participant characteristics by COVID-19 case status**

|  | **COVID-19 +**  **cases (n=22)*** | **COVID-19 - controls (n=44)*** | **Overall (N=66)** |
| --- | --- | --- | --- |
| **Age (years)^†^** | 45.0 [20.0, 66.0] | 43.0 [18.0, 72.0] | 44.0 [18.0, 72.0] |
| **Sex (biological)** |  |  |  |
| Male | 11 (50.0%) | 22 (50.0%) | 33 (50.0%) |
| Female | 10 (45.5%) | 20 (45.5%) | 30 (45.5%) |
| Prefer not to say | 1 (4.5%) | 2 (4.5%) | 3 (4.5%) |
| **Hispanic ethnicity** |  |  |  |
| No | 19 (86.4%) | 35 (79.5%) | 54 (81.8%) |
| Yes | 2 (9.1%) | 7 (15.9%) | 9 (13.6%) |
| Prefer not to say | 1 (4.5%) | 2 (4.5%) | 3 (4.5%) |
| **Race** |  |  |  |
| American Indian or Alaska Native | 1 (4.5%) | 3 (6.8%) | 4 (6.1%) |
| Asian | 0 (0%) | 3 (6.8%) | 3 (4.5%) |
| Black or African American | 10 (45.5%) | 16 (36.4%) | 26 (39.4%) |
| Native Hawaiian or other Pacific Islander | 1 (4.5%) | 3 (6.8%) | 4 (6.1%) |
| White | 4 (18.2%) | 10 (22.7%) | 14 (21.2%) |
| Multiple Races | 2 (9.1%) | 2 (4.5%) | 4 (6.1%) |
| Other | 2 (9.1%) | 0 (0.0%) | 2 (3.0%) |
| Prefer not to say | 2 (9.1%) | 7 (15.9%) | 9 (13.6%) |
| **Language** |  |  |  |
| English | 19 (86.4%) | 43 (97.7%) | 62 (93.9%) |
| Spanish | 2 (9.1%) | 1 (2.3%) | 3 (4.5%) |
| Tigrinya | 1 (4.5%) | 0 (0.0%) | 1 (1.5%) |
| **Education** |  |  |  |
| Less than high school graduate | 4 (18.2%) | 4 (9.1%) | 8 (12.1%) |
| Graduated high school/obtained GED | 7 (31.8%) | 12 (27.3%) | 19 (28.8%) |
| Some college**^‡^** | 3 (13.6%) | 14 (31.8%) | 17 (25.8%) |
| Bachelor's or advanced degree | 1 (4.5%) | 13 (29.5%) | 14 (21.2%) |
| Prefer not to say | 1 (4.5%) | 1 (2.3%) | 2 (3.0%) |
| Missing | 6 (27.3%) | 0 (0%) | 6 (9.1%) |
| **Employed** |  |  |  |
| No | 13 (59.1%) | 23 (52.3%) | 36 (54.5%) |
| Yes | 3 (13.6%) | 21 (47.7%) | 24 (36.4%) |
| Missing | 6 (27.3%) | 0 (0%) | 6 (9.1%) |
| **Income** |  |  |  |
| ≤ $25,000 | 10 (45.5%) | 25 (56.8%) | 35 (53.0%) |
| > $25,000 | 1 (4.5%) | 11 (25.0%) | 12 (18.2%) |
| Don't know or prefer not to say | 5 (22.7%) | 8 (18.2%) | 13 (19.7%) |
| Missing | 6 (27.3%) | 0 (0.0%) | 6 (9.1%) |
| **Insurance** |  |  |  |
| Private | 0 (0.0%) | 11 (25.0%) | 11 (16.7%) |
| Government | 15 (68.2%) | 24 (54.5%) | 39 (59.1%) |
| None | 0 (0.0%) | 7 (15.9%) | 7 (10.6%) |
| Prefer not to say | 1 (4.5%) | 2 (4.5%) | 3 (4.5%) |
| Missing | 6 (27.3%) | 0 (0.0%) | 6 (9.1%) |
| **Duration of homelessness** |  |  |  |
| 6 months or less | 2 (9.1%) | 10 (22.7%) | 12 (18.2%) |
| 7-12 months | 1 (4.5%) | 7 (15.9%) | 8 (12.1%) |
| 13-24 months | 4 (18.2%) | 3 (6.8%) | 7 (10.6%) |
| Over 24 months (2 years) | 8 (36.4%) | 9 (20.5%) | 17 (25.8%) |
| Prefer Not to Say | 1 (4.5%) | 2 (4.5%) | 3 (4.5%) |
| Missing | 6 (27.3%) | 13 (29.5%) | 19 (28.8%) |
| **Shelter Type** |  |  |  |
| Mixed gender, ≥ 18 years | 11 (50.0%) | 16 (36.4%) | 27 (40.9%) |
| Mixed gender, 18 - 25 years | 0 (0.0%) | 3 (6.8%) | 3 (4.5%) |
| Female, ≥ 18 years | 0 (0.0%) | 4 (9.1%) | 4 (6.1%) |
| Male, ≥ 18 years | 2 (9.1%) | 4 (9.1%) | 6 (9.1%) |
| Male, ≥ 50 years | 1 (4.5%) | 3 (6.8%) | 4 (6.1%) |
| Mixed gender, all ages | 8 (36.4%) | 14 (31.8%) | 22 (33.3%) |
| **Any comorbidities^§^** | 6 (27.3%) | 13 (29.5%) | 19 (28.8%) |
| **Smoking status** |  |  |  |
| None | 12 (54.5%) | 26 (59.1%) | 38 (57.6%) |
| Tobacco products | 9 (40.9%) | 16 (36.4%) | 25 (37.9%) |
| Prefer not to say | 1 (4.5%) | 2 (4.5%) | 3 (4.5%) |
| **Any symptoms at baseline** | 5 (22.7%) | 6 (13.6%) | 11 (16.7%) |
| **Follow-up time since enrollment (days)^†^** | 72.0 [35.0, 183] | 157 [96.0, 223] | 126 [35.0, 223] |
| **Follow-up season^‖^** |  |  |  |
| Fall | 1 (4.5%) | 29 (65.9%) | 30 (45.5%) |
| Spring | 0 (0.0%) | 15 (34.1%) | 15 (22.7%) |
| Winter | 3 (13.6%) | 0 (0.0%) | 3 (4.5%) |
| Summer | 18 (81.8%) | 0 (0.0%) | 18 (27.3%) |
| **Any symptoms at follow-up^a^** | 9 (40.9%) | 12 (27.3%) | 21 (31.8%) |

* + = positive; - = negative

**^†^** Median [Min, Max]

**^‡^** Some college includes: vocational training, associate's degree

**^§^** Any comorbidities includes: asthma, blood disorders, cancer, chronic obstructive pulmonary disease or emphysema, immunosuppression, liver disease, heart disease, diabetes, neurologic conditions, or aspirin therapy.

**^‖^** Seasons defined by astronomical season in the Northern Hemisphere (using equinox and solstice dates)

^a^ Any symptoms at follow-up represents a single follow-up time point between day 30-225 post-enrollment. If a COVID-19 case had more than one follow-up survey completed between day 30-225 post-enrollment, the survey from the median timepoint was selected and used.

**Table S3. Participant characteristics of COVID-19 cases: reached vs. unable to reach**

|  |  |  |
| --- | --- | --- |
|  | **Reached**  **(n=22)** | **Unable to reach (n=31)*** |
| **Age (years)^†^** | 45.0 [20.0, 66.0] | 40.0 [19.0, 83.0] |
| **Sex (biological)** |  |  |
| Male | 11 (50.0%) | 18 (58.1%) |
| Female | 10 (45.5%) | 11 (35.5%) |
| Prefer not to say | 1 (4.5%) | 2 (6.5%) |
| **Hispanic ethnicity** |  |  |
| No | 19 (86.4%) | 28 (90.3%) |
| Yes | 2 (9.1%) | 1 (3.2%) |
| Prefer not to say | 1 (4.5%) | 2 (6.5%) |
| **Race** |  |  |
| American Indian or Alaska Native | 1 (4.5%) | 3 (9.7%) |
| Asian | 0 (0%) | 0 (0.0%) |
| Black or African American | 10 (45.5%) | 15 (48.4%) |
| Native Hawaiian or other Pacific Islander | 1 (4.5%) | 0 (0.0%) |
| White | 4 (18.2%) | 5 (16.1%) |
| Multiracial | 2 (9.1%) | 3 (9.7%) |
| Other | 2 (9.1%) | 0 (0.0%) |
| Prefer not to say | 2 (9.1%) | 5 (16.1%) |
| **Language** |  |  |
| English | 19 (86.4%) | 28 (90.3%) |
| Spanish | 2 (9.1%) | 0 (0.0%) |
| Other | 1 (4.5%) | 3 (9.6%) |
| **Education** |  |  |
| Less than high school graduate | 4 (18.2%) | 7 (22.6%) |
| Graduated high school/obtained GED | 7 (31.8%) | 7 (22.6%) |
| Some college^‡^ | 3 (13.6%) | 6 (19.4%) |
| Bachelor's or advanced degree | 1 (4.5%) | 2 (6.5%) |
| Prefer not to say | 1 (4.5%) | 3 (9.7%) |
| Missing | 6 (27.3%) | 6 (19.4%) |
| **Employed** |  |  |
| No | 13 (59.1%) | 19 (61.3%) |
| Yes | 3 (13.6%) | 5 (16.1%) |
| Missing | 6 (27.3%) | 7 (22.6%) |
| **Income** |  |  |
| ≤ $25,000 | 10 (45.5%) | 11 (35.5%) |
| > $25,000 | 1 (4.5%) | 2 (6.5%) |
| Don't know or prefer not to say | 5 (22.7%) | 12 (38.7%) |
| Missing | 6 (27.3%) | 6 (19.4%) |
| **Insurance** |  |  |
| Private | 0 (0.0%) | 2 (6.5%) |
| Government | 15 (68.2%) | 19 (61.3%) |
| None | 0 (0.0%) | 1 (3.2%) |
| Prefer not to say | 1 (4.5%) | 2 (6.5%) |
| Missing | 6 (27.3%) | 7 (22.6%) |
| **Duration of homelessness** |  |  |
| 6 months or less | 2 (9.1%) | 6 (19.4%) |
| 7-12 months | 1 (4.5%) | 0 (0.0%) |
| 13-24 months | 4 (18.2%) | 3 (9.7%) |
| Over 24 months (2 years) | 8 (36.4%) | 10 (32.3%) |
| Prefer Not to Say | 1 (4.5%) | 3 (9.7%) |
| Missing | 6 (27.3%) | 9 (29.0%) |
| **Shelter Type** |  |  |
| Mixed gender, ≥ 18 years | 11 (50.0%) | 19 (61.3%) |
| Mixed gender, 18 - 25 years | 0 (0.0%) | 1 (3.2%) |
| Female, ≥ 18 years | 0 (0.0%) | 2 (6.5%) |
| Male, ≥ 18 years | 2 (9.1%) | 2 (6.5%) |
| Male, ≥ 50 years | 1 (4.5%) | 0 (0.0%) |
| Mixed gender, all ages | 8 (36.4%) | 7 (22.6%) |
| **Any comorbidities^§^** | 6 (27.3%) | 4 (22.6%) |
| **Smoking status** |  |  |
| None | 12 (54.5%) | 12 (38.7%) |
| Tobacco products or electronic cigarettes | 9 (40.9%) | 15 (48.4%) |
| Prefer not to say | 1 (4.5%) | 2 (6.5%) |
| **Any symptoms at enrollment** | 5 (22.7%) | 7 (22.6%) |
| **Any phone number provided** | 17 (77.3%) | 15 (48.4%) |

* Cases in “Unable to reach (n=31)” include those who were deceased (n=1), declined to participate (n=8), and unable to reach (n=22)

^†^ Median [Min, Max]

^‡^ Some college includes: vocational training, associate's degree

**^§^** Any comorbidities includes: asthma, blood disorders, cancer, chronic obstructive pulmonary disease or emphysema, immunosuppression, liver disease, heart disease, diabetes, neurologic conditions, or aspirin therapy.

**Table S4. Participant characteristics of COVID-19 controls: reached vs. unable to reach**

|  |  |  |
| --- | --- | --- |
|  | **Reached**  **(n=44)** | **Unable to reach (n=60)*** |
| **Age (years)^†^** | 43.0 [18.0, 72.0] | 43.0 [19.0, 71.0] |
| **Sex (biological)** |  |  |
| Male | 22 (50.0%) | 36 (60.0%) |
| Female | 20 (45.5%) | 23 (38.3%) |
| Prefer not to say | 2 (4.5%) | 1 (1.7%) |
| **Hispanic ethnicity** |  |  |
| No | 35 (79.5%) | 48 (80.0%) |
| Yes | 7 (15.9%) | 10 (16.7%) |
| Prefer not to say | 2 (4.5%) | 2 (3.3%) |
| **Race** |  |  |
| American Indian or Alaska Native | 3 (6.8%) | 3 (5.0%) |
| Asian | 3 (6.8%) | 2 (3.3%) |
| Black or African American | 16 (36.4%) | 20 (33.3%) |
| Native Hawaiian or other Pacific Islander | 3 (6.8%) | 4 (6.7%) |
| White | 10 (22.7%) | 16 (26.7%) |
| Multiracial | 2 (4.5%) | 4 (6.7%) |
| Other | 0 (0.0%) | 0 (0.0%) |
| Prefer not to say | 7 (15.9%) | 11 (18.3%) |
| **Language** |  |  |
| English | 43 (97.7%) | 56 (93.3%) |
| Spanish | 1 (2.3%) | 3 (5.0%) |
| Other | 0 (0.0%) | 1 (1.7%) |
| **Education** |  |  |
| Less than high school graduate | 4 (9.1%) | 13 (21.7%) |
| Graduated high school/obtained GED | 12 (27.3%) | 15 (25.0%) |
| Some college^‡^ | 14 (31.8%) | 22 (36.7%) |
| Bachelor's or advanced degree | 13 (29.5%) | 7 (11.7%) |
| Prefer not to say | 1 (2.3%) | 3 (5.0%) |
| **Employed** |  |  |
| No | 23 (52.3%) | 44 (73.3%) |
| Yes | 21 (47.7%) | 16 (26.7%) |
| **Income** |  |  |
| ≤ $25,000 | 25 (56.8%) | 38 (63.3%) |
| > $25,000 | 11 (25.0%) | 6 (10.0%) |
| Don't know or prefer not to say | 8 (18.2%) | 16 (26.7%) |
| **Insurance** |  |  |
| Private | 11 (25.0%) | 7 (11.7%) |
| Government | 24 (54.5%) | 43 (71.7%) |
| None | 7 (15.9%) | 9 (15.0%) |
| Prefer not to say | 2 (4.5%) | 1 (1.7%) |
| **Duration of homelessness** |  |  |
| 6 months or less | 10 (22.7%) | 15 (25.0%) |
| 7-12 months | 7 (15.9%) | 7 (11.7%) |
| 13-24 months | 3 (6.8%) | 9 (15.0%) |
| Over 24 months (2 years) | 9 (20.5%) | 16 (26.7%) |
| Prefer Not to Say | 2 (4.5%) | 1 (1.7%) |
| Missing | 13 (29.5%) | 12 (20.0%) |
| **Shelter Type** |  |  |
| Mixed gender, ≥ 18 years | 16 (36.4%) | 25 (41.7%) |
| Mixed gender, 18 - 25 years | 3 (6.8%) | 3 (5.0%) |
| Female, ≥ 18 years | 4 (9.1%) | 3 (5.0%) |
| Male, ≥ 18 years | 4 (9.1%) | 4 (6.7%) |
| Male, ≥ 50 years | 3 (6.8%) | 3 (5.0%) |
| Mixed gender, all ages | 14 (31.8%) | 22 (36.7%) |
| **Any comorbidities^§^** | 13 (29.5%) | 16 (26.7%) |
| **Smoking status** |  |  |
| None | 26 (59.1%) | 28 (46.7%) |
| Tobacco products or electronic cigarettes | 16 (36.4%) | 31 (51.7%) |
| Prefer not to say | 2 (4.5%) | 1 (1.7%) |
| **Any symptoms at enrollment** | 6 (13.6%) | 7 (11.7%) |
| **Any phone number provided** | 44 (100%) | 60 (100%) |

* Controls in “Unable to reach (n=60)” include those who were deceased (n=1), declined to participate (n=8), and unable to reach (n=51)

^†^ Median [Min, Max]

^‡^ Some college includes: vocational training, associate's degree

**^§^** Any comorbidities includes: asthma, blood disorders, cancer, chronic obstructive pulmonary disease or emphysema, immunosuppression, liver disease, heart disease, diabetes, neurologic conditions, or aspirin therapy.

**Figure S1. Study flow diagram**

**
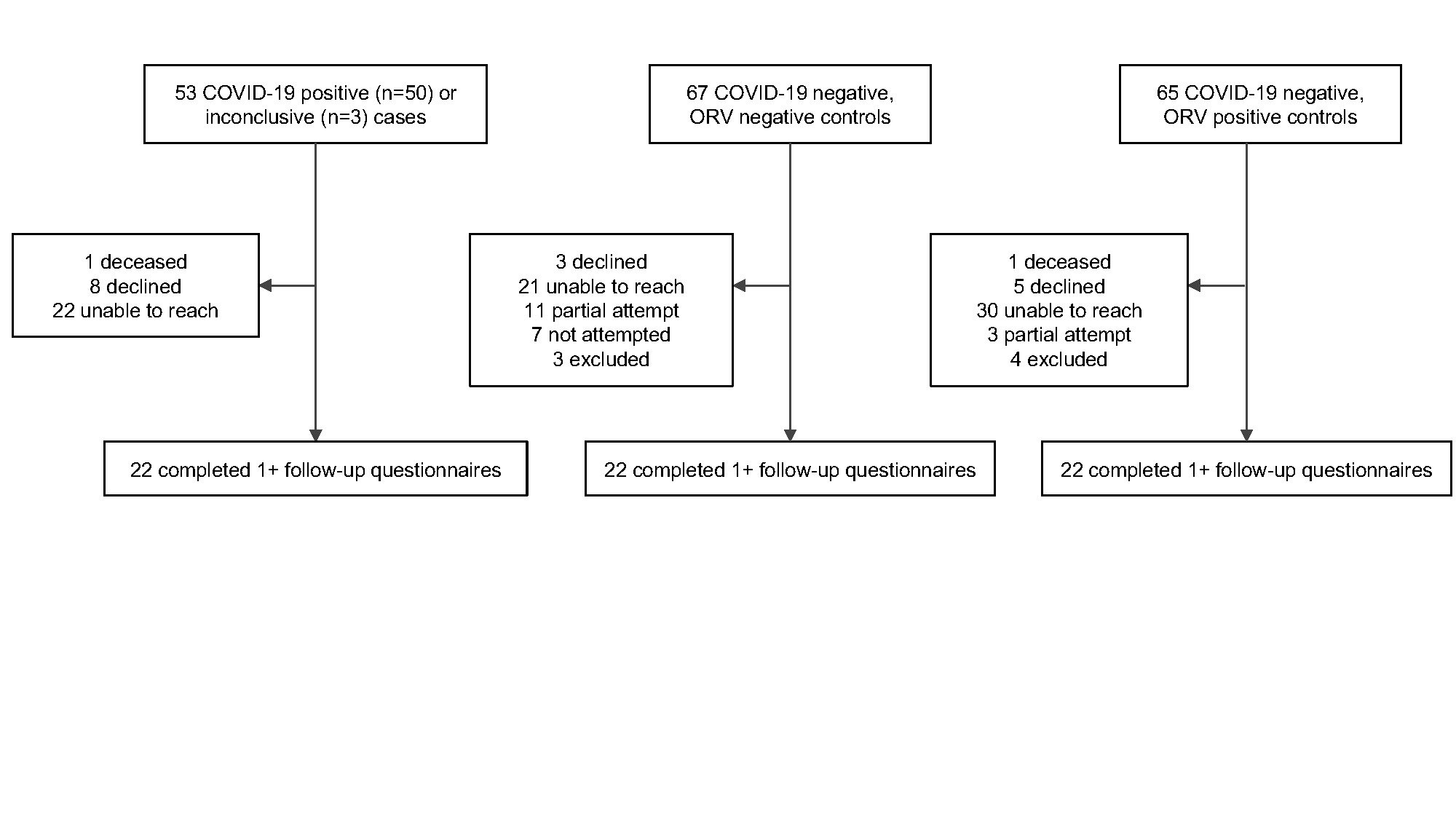
**

**Figure S2. Symptoms reported at enrollment and follow-up by COVID-19 and Other Respiratory Virus (ORV) case status**

| **COVID-19-positive cases**  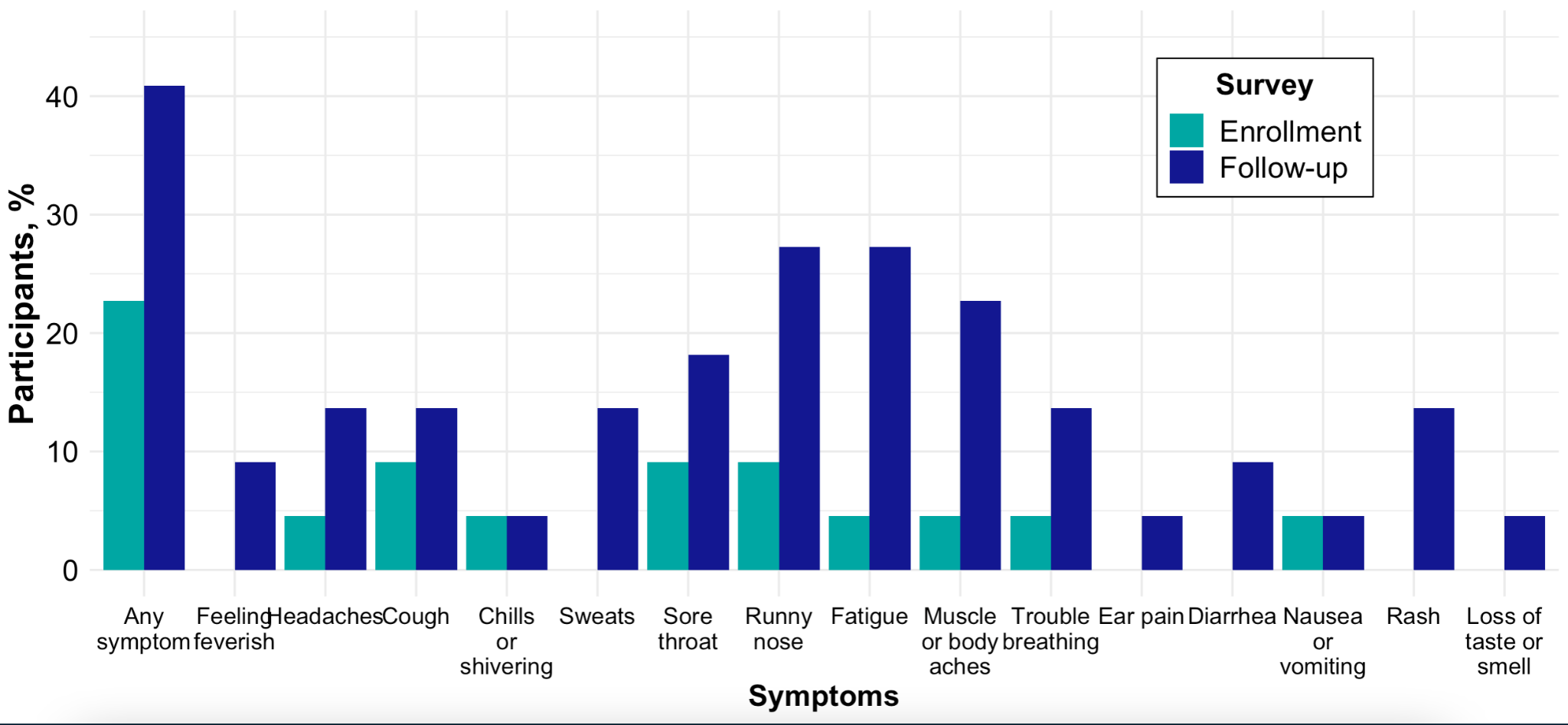 |
| --- |
| **COVID-19-negative, ORV-positive controls**  *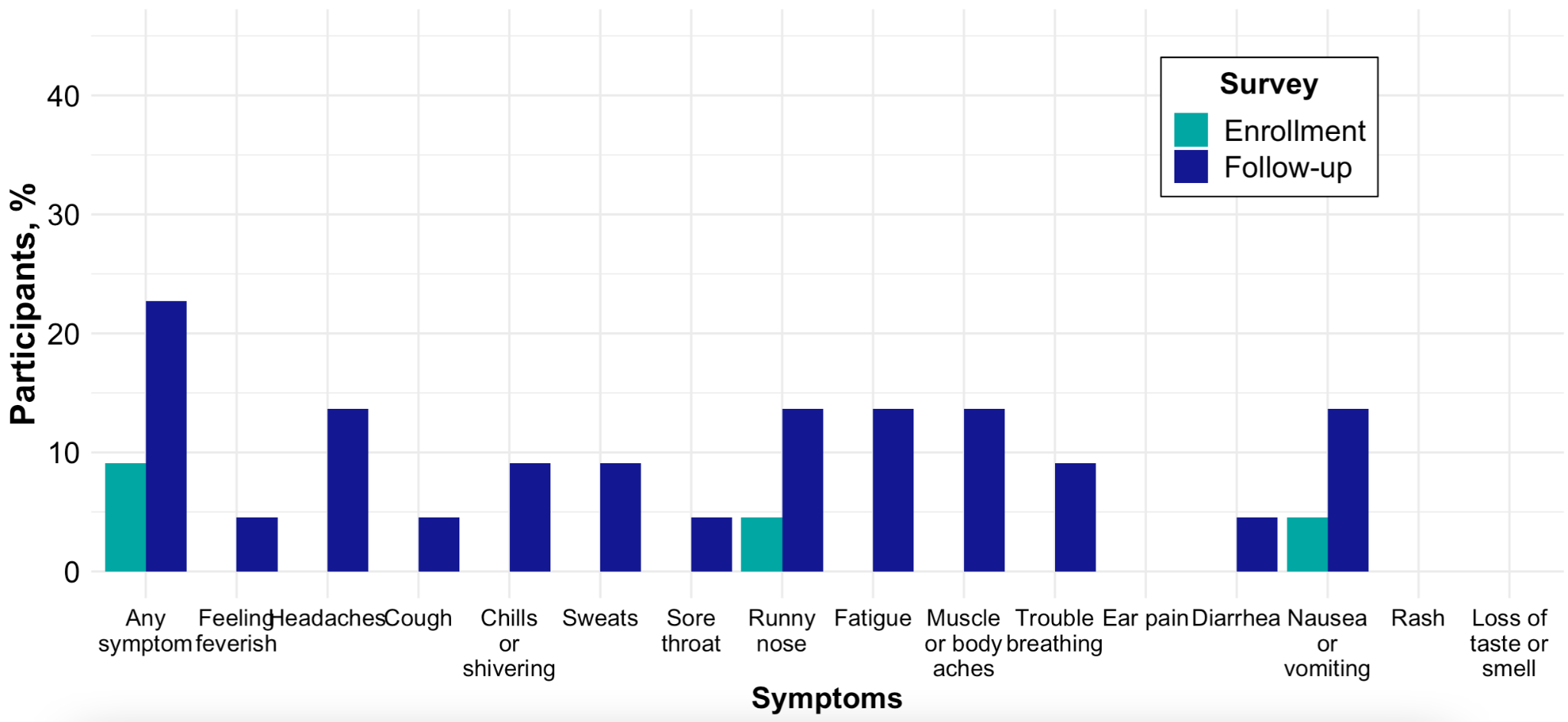* |
| **COVID-19-negative, ORV-negative controls**  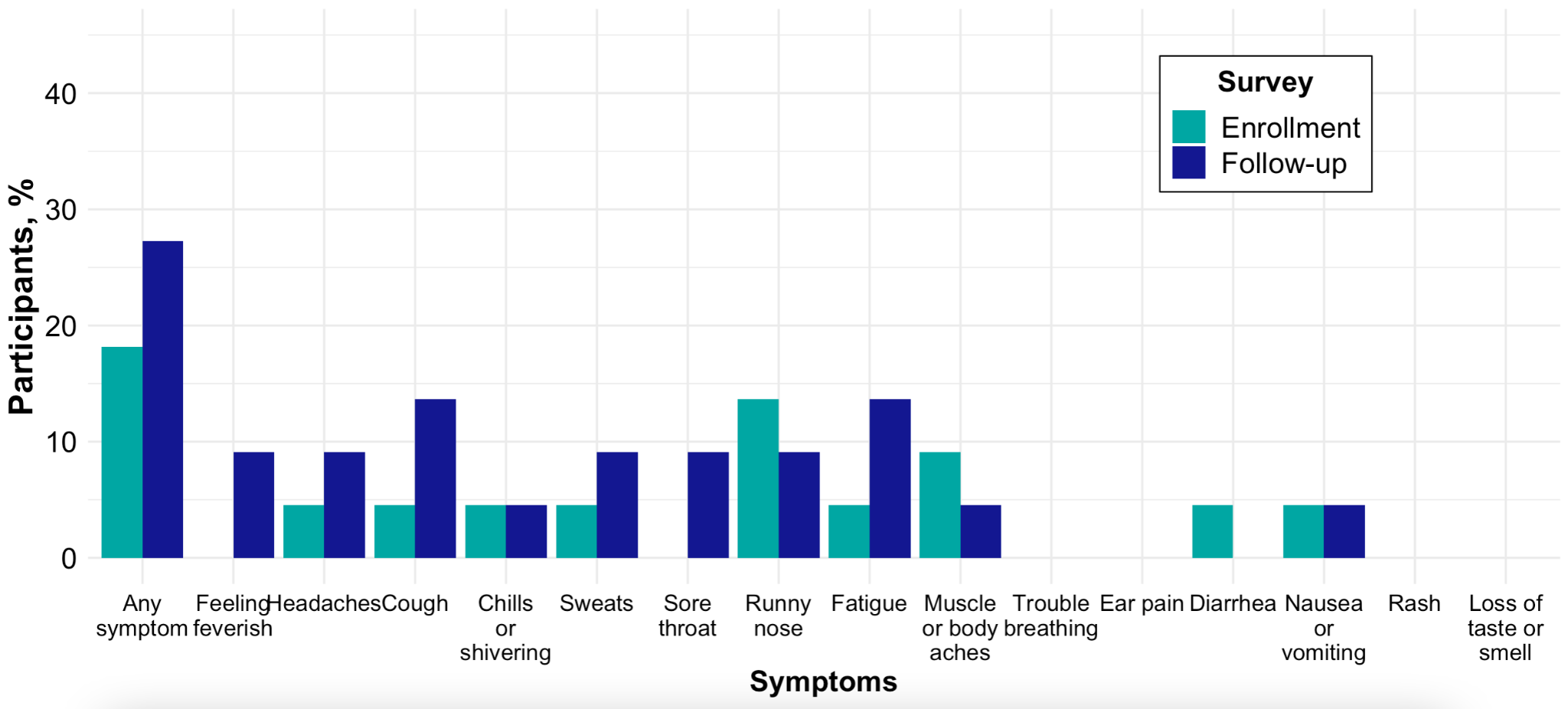 |

**Figure S3. Dot plot of symptoms and impact on daily activity at follow-up by COVID-19 case status**

**COVID-19-positive cases (n=22)**

**
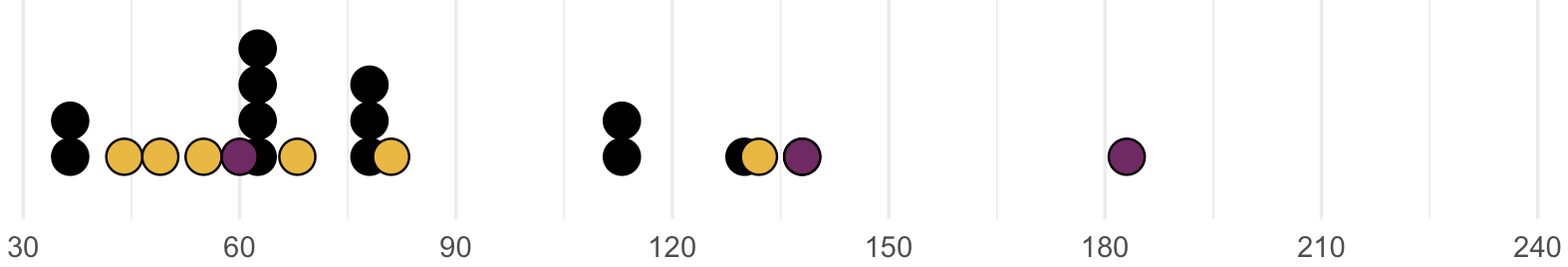
**

**COVID-19-negative, ORV-positive controls
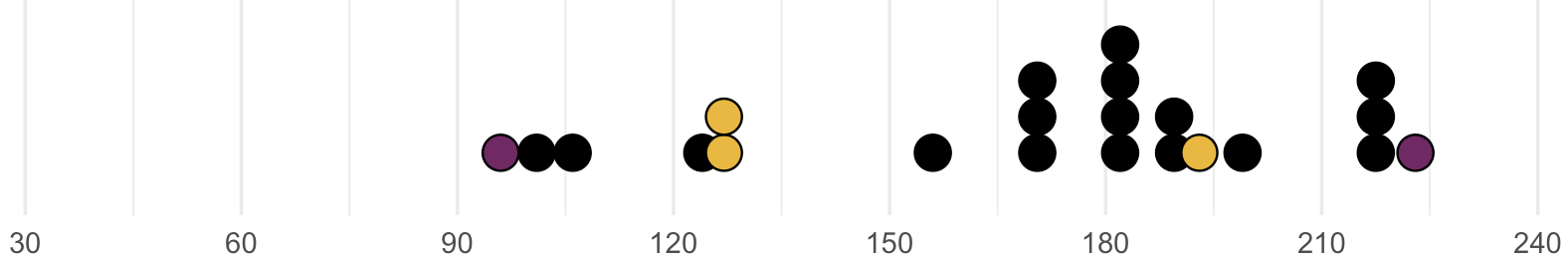
**

**COVID-19-negative, ORV-negative controls
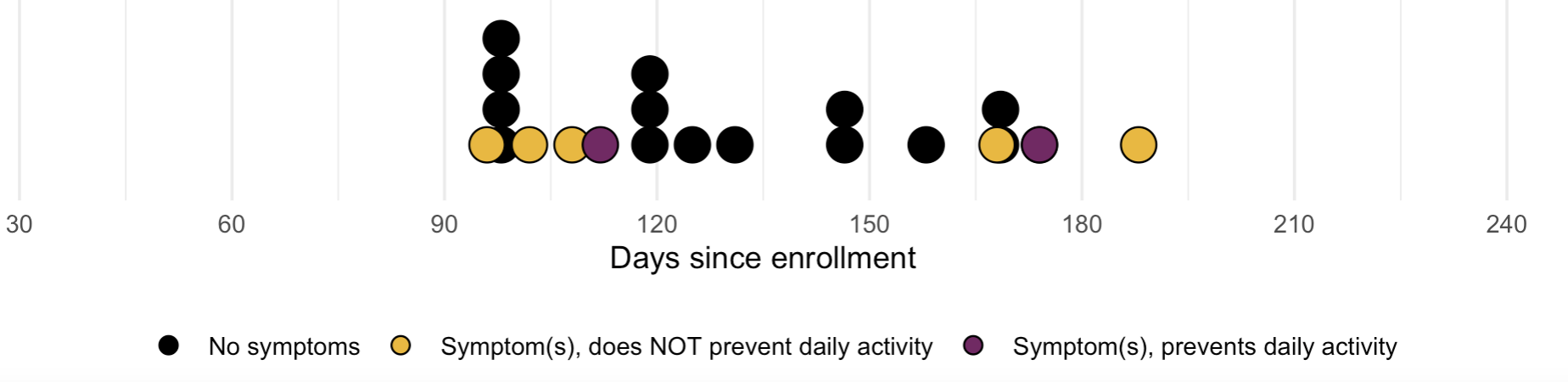
**
