# Supplementary material for: Burden of long COVID among adults experiencing sheltered homelessness: a longitudinal cohort study in King County, WA between September 2020—April 2022
Source: BMC Public Health. 2023 Jun 6;23:1079. doi: 10.1186/s12889-023-16026-7 (PMC10241609; doi:10.1186/s12889-023-16026-7)
Supplement: Supplementary file 2 — Additional file 2: Appendix 2. Enrollment Questionnaire. [file 12889_2023_16026_MOESM2_ESM.docx]

1. Date and Time: MM/DD/YYYY HH:MM [AM/PM]
2. Shelter location: ___________________
3. Are you a shelter staff member?
   - Yes
   - No
4. What is your preferred language for your study participation?
   - English
   - Spanish
   - Amharic
   - Tigrinya
   - Ngala
   - Marshallese
   - Other, please specify: ___________________
5. Enter your birthday: MM/DD/YYYY
6. Enter your phone number: ___________________
7. Have you experienced any of these new or worsening symptoms in the last seven days?* Select all that apply.
   - Feeling feverish
   - Headaches
   - Cough
   - Chills or shivering
   - Sweats
   - Sore throat or itchy/scratchy throat
   - Runny / stuffy nose
   - Feeling more tired than usual
   - Muscle or body aches
   - Increased trouble with breathing
   - Ear pain or ear discharge
   - Diarrhea
   - Nausea or vomiting
   - Rash
   - Loss of smell or taste
   - None of the above🡪*Skip to question 11*

*NOTE: We want to know if you have NEW or WORSE health problems. For example, some people always cough and we want to know if and when your cough GOT WORSE.

1. When did these symptoms you listed become new or worsening?
   - Half a day ago
   - Half a day - 1 day ago
   - 1 - 1.5 days ago
   - 1.5 - 2 days ago
   - 3 days ago
   - 4 days ago
   - 5 or more days ago
   - I don't have any new or worsening symptoms 🡪*Skip to question 11*
2. How severe are your symptoms? Select the level of discomfort you felt at the worst point.
   1. Feeling feverish
      - Mild (does not interfere with activity)
      - Moderate (interferes with daily activity)
      - Severe (prevents daily activity)
      - Requiring emergency department visit or hospitalization
   2. Headaches
      - Mild (does not interfere with activity)
      - Moderate (interferes with daily activity)
      - Severe (prevents daily activity)
      - Requiring emergency department visit or hospitalization
   3. Cough
      - Mild (does not interfere with activity)
      - Moderate (interferes with daily activity)
      - Severe (prevents daily activity)
      - Requiring emergency department visit or hospitalization
   4. Chills or shivering
      - Mild (does not interfere with activity)
      - Moderate (interferes with daily activity)
      - Severe (prevents daily activity)
      - Requiring emergency department visit or hospitalization
   5. Sweats
      - Mild (does not interfere with activity)
      - Moderate (interferes with daily activity)
      - Severe (prevents daily activity)
      - Requiring emergency department visit or hospitalization
   6. Sore throat or itchy/scratchy throat
      - Mild (does not interfere with activity)
      - Moderate (interferes with daily activity)
      - Severe (prevents daily activity)
      - Requiring emergency department visit or hospitalization
   7. Runny/stuffy nose
      - Mild (does not interfere with activity)
      - Moderate (interferes with daily activity)
      - Severe (prevents daily activity)
      - Requiring emergency department visit or hospitalization
   8. Feeling more tired than usual
      - Mild (does not interfere with activity)
      - Moderate (interferes with daily activity)
      - Severe (prevents daily activity)
      - Requiring emergency department visit or hospitalization
   9. Muscle or body aches
      - Mild (does not interfere with activity)
      - Moderate (interferes with daily activity)
      - Severe (prevents daily activity)
      - Requiring emergency department visit or hospitalization
   10. Increase trouble with breathing
       - Mild (does not interfere with activity)
       - Moderate (interferes with daily activity)
       - Severe (prevents daily activity)
       - Requiring emergency department visit or hospitalization
   11. Ear pain or ear discharge
       - Mild (does not interfere with activity)
       - Moderate (interferes with daily activity)
       - Severe (prevents daily activity)
       - Requiring emergency department visit or hospitalization
   12. Diarrhea
       - Mild (does not interfere with activity)
       - Moderate (interferes with daily activity)
       - Severe (prevents daily activity)
       - Requiring emergency department visit or hospitalization
   13. Nausea or vomiting
       - Mild (does not interfere with activity)
       - Moderate (interferes with daily activity)
       - Severe (prevents daily activity)
       - Requiring emergency department visit or hospitalization
   14. Rash
       - Mild (does not interfere with activity)
       - Moderate (interferes with daily activity)
       - Severe (prevents daily activity)
       - Requiring emergency department visit or hospitalization
   15. Loss of smell or taste
       - Mild (does not interfere with activity)
       - Moderate (interferes with daily activity)
       - Severe (prevents daily activity)
       - Requiring emergency department visit or hospitalization
3. When your recent illness was at its worst, how did it affect your ability to do your regular activities (work, school, etc.)?
   - Not at all
   - A little bit
   - Somewhat
   - Quite a bit
   - Very much
4. Which of the following daily activities have been impacted by your current illness? Select all that apply.
   - Work
   - School
   - Running errands
   - Exercising
   - Socializing
   - Looking for work
   - Ability to take care of myself and/or family
   - None of the above/ my activities have not been impacted
   - Prefer not to say
5. Have you previously been tested for COVID-19?
   - Yes
   - No🡪*Skip to question 14*
6. Have you ever tested positive for COVID-19?
   - Yes
   - No 🡪*Skip to question 14*
7. What was the date of your most recent positive COVID-19 swab test?
   - MM/DD/YYYY
8. What is your sex?
   - Male
   - Female
   - Other, please specify: ___________________
   - Prefer not to say
9. Are you Hispanic or Latino?
   - Yes
   - No
   - Prefer not to say
10. How would you describe your race? Select all that apply.
    - American Indian or Alaska Native
    - Asian
    - Native Hawaiian or other Pacific Islander
    - Black or African American
    - White
    - Other
    - Prefer not to say
11. Have you ever been told by a healthcare provider that you have one of the following medical conditions? Select all that apply.
    - Asthma or reactive airway disease
    - Blood disorders (e.g., sickle cell)
    - COPD/ emphysema
    - Chronic bronchitis
    - Cancer
    - Diabetes
    - Heart disease (heart failure or heart attack)
    - Immunosuppression (by medication or disease)
    - Liver disease
    - None of these conditions
    - Do not know
    - Prefer not to say
12. What is the highest level of education you have completed?
    - Less than high school graduate
    - Graduated high school/obtained GED
    - Some college (including vocational training,associate's degree)
    - Bachelor's degree
    - Advanced degree
    - Prefer not to say
13. Please choose the range that best represents your household income last year (before taxes). If you are still considered a "dependent" for tax purposes, choose the range that describes your parent/legal guardian's household income.
    - Less than or equal to $25,000
    - Between $25 and 50 thousand ($25,001 to $50,000)
    - Between $50 and 75 thousand ($50,001 to $75,000)
    - Between $75 and 100 thousand ($75,001 to$100,000)
    - Between $100 and 125 thousand ($100,001 to$125,000)
    - Between $125 and 150 thousand ($125,001 to$150,000)
    - Over $150,000
    - Don't know
    - Prefer not to say
14. What type of health insurance do you have? Select all that apply.
    - Private (provided by employer and/or purchased)
    - Government (Medicare/Medicaid)
    - Other
    - None
    - Prefer not to say
15. How long have you been experiencing homelessness? We consider homelessness to be living without permanent housing(which may include staying with friends, in a hotel, shelter, church, on the streets, in a car, or in any other unstable or non-permanent situation).
    - 6 months or less
    - 7-12 months
    - 13-24 months
    - Over 24 months (2 years)
    - Do Not Know
    - Prefer Not to Say
16. Are you currently employed?
    - Yes
    - No
17. Do you use any of the following products (either indoors or outdoors)? Select all that apply.
    - Tobacco products (e.g. cigarettes, cigars, pipes)
    - Electronic cigarettes/vapor pens
    - None of the above
    - Prefer not to say
