# Supplementary material for: Burden of long COVID among adults experiencing sheltered homelessness: a longitudinal cohort study in King County, WA between September 2020—April 2022
Source: BMC Public Health. 2023 Jun 6;23:1079. doi: 10.1186/s12889-023-16026-7 (PMC10241609; doi:10.1186/s12889-023-16026-7)
Supplement: Supplementary file 4 — Additional file 4. Appendix 4. COVID-19 Control Follow-up Questionnaire. [file 12889_2023_16026_MOESM4_ESM.docx]

BEFORE STARTING SURVEY: Have you tested positive for the virus that causes COVID-19 since [enrollment date]?

- - No
  - Yes 🡪If selected, DO NOT COMPLETE SURVEY AS INELIGIBLE TO BE A CONTROL

1. Date and Time: MM/DD/YYYY HH:MM [AM/PM]
2. In the last seven days, have you experienced any of the following symptoms that were new or worsening symptoms since [enrollment date]?* Select all that apply.
   - Feeling feverish
   - Headaches
   - Cough
   - Chills or shivering
   - Sweats
   - Sore throat or itchy/scratchy throat
   - Runny / stuffy nose
   - Feeling more tired than usual
   - Muscle or body aches
   - Increased trouble with breathing
   - Ear pain or ear discharge
   - Diarrhea
   - Nausea or vomiting
   - Rash
   - Loss of smell or taste
   - None of the above🡪*Skip to question 4*

**NOTE: on day 5 survey modify to* “Have you experienced any of the following new or worsening symptoms since [enrollment date]?”

1. When did these symptoms you listed become new or worsening?
   - Half a day ago
   - Half a day - 1 day ago
   - 1 - 1.5 days ago
   - 1.5 - 2 days ago
   - 3 days ago
   - 4 days ago
   - 5 or more days ago
   - I don't have any new or worsening symptoms 🡪*Skip to question 4*
2. How severe have your symptoms been in the last seven days?* Select the level of discomfort you felt at the worst point.
   1. Feeling feverish
      - Mild (does not interfere with activity)
      - Moderate (interferes with daily activity)
      - Severe (prevents daily activity)
      - Requiring emergency department visit or hospitalization
   2. Headaches
      - Mild (does not interfere with activity)
      - Moderate (interferes with daily activity)
      - Severe (prevents daily activity)
      - Requiring emergency department visit or hospitalization
   3. Cough
      - Mild (does not interfere with activity)
      - Moderate (interferes with daily activity)
      - Severe (prevents daily activity)
      - Requiring emergency department visit or hospitalization
   4. Chills or shivering
      - Mild (does not interfere with activity)
      - Moderate (interferes with daily activity)
      - Severe (prevents daily activity)
      - Requiring emergency department visit or hospitalization
   5. Sweats
      - Mild (does not interfere with activity)
      - Moderate (interferes with daily activity)
      - Severe (prevents daily activity)
      - Requiring emergency department visit or hospitalization
   6. Sore throat or itchy/scratchy throat
      - Mild (does not interfere with activity)
      - Moderate (interferes with daily activity)
      - Severe (prevents daily activity)
      - Requiring emergency department visit or hospitalization
   7. Runny/stuffy nose
      - Mild (does not interfere with activity)
      - Moderate (interferes with daily activity)
      - Severe (prevents daily activity)
      - Requiring emergency department visit or hospitalization
   8. Feeling more tired than usual
      - Mild (does not interfere with activity)
      - Moderate (interferes with daily activity)
      - Severe (prevents daily activity)
      - Requiring emergency department visit or hospitalization
   9. Muscle or body aches
      - Mild (does not interfere with activity)
      - Moderate (interferes with daily activity)
      - Severe (prevents daily activity)
      - Requiring emergency department visit or hospitalization
   10. Increase trouble with breathing
       - Mild (does not interfere with activity)
       - Moderate (interferes with daily activity)
       - Severe (prevents daily activity)
       - Requiring emergency department visit or hospitalization
   11. Ear pain or ear discharge
       - Mild (does not interfere with activity)
       - Moderate (interferes with daily activity)
       - Severe (prevents daily activity)
       - Requiring emergency department visit or hospitalization
   12. Diarrhea
       - Mild (does not interfere with activity)
       - Moderate (interferes with daily activity)
       - Severe (prevents daily activity)
       - Requiring emergency department visit or hospitalization
   13. Nausea or vomiting
       - Mild (does not interfere with activity)
       - Moderate (interferes with daily activity)
       - Severe (prevents daily activity)
       - Requiring emergency department visit or hospitalization
   14. Rash
       - Mild (does not interfere with activity)
       - Moderate (interferes with daily activity)
       - Severe (prevents daily activity)
       - Requiring emergency department visit or hospitalization
   15. Loss of smell or taste
       - Mild (does not interfere with activity)
       - Moderate (interferes with daily activity)
       - Severe (prevents daily activity)
       - Requiring emergency department visit or hospitalization
3. Since [enrollment date] have you newly required supplemental oxygen?
   - No
   - Yes and I continue to need supplemental oxygen
   - Yes, but I don't need it anymore
4. Thinking about your symptoms in the last seven* days, do you feel better, as sick, or worse than when you were tested for COVID-19 on [enrollment date]?
   - Better
   - As sick
   - Worse
5. Have the symptoms you mentioned above affected your ability to do your regular activities (work, school, etc.)?
   - Not at all
   - A little bit
   - Somewhat
   - Quite a bit
   - Very much
6. Which of the following daily activities have been affected so far? Select all that apply.
   - Work
   - School
   - Running errands
   - Exercising
   - Socializing
   - Looking for work
   - Ability to take care of myself and/or family
   - Accessing overnight shelter services
   - Accessing other services (housing, laundry, food, case management)
   - None of the above/ my activities have not been impacted
   - Prefer not to say
7. Did any of the following occur in the last week because of your symptoms? Select all that apply.
   - I missed work *🡪 If selected, continue to Question 9. If not selected, skip to question 10.*
   - I worked from home
   - I worked fewer hours than usual.
   - I lost my job
   - None of the above
8. How many days were you not able to go to work?
9. Did your symptoms keep you from doing any of the following? Select all that apply.
   - Attending class
   - Going to work
   - Studying
   - Performing well on an exam or written assignment
   - None of the above/ My activities have not been impacted
10. Have you received any clinical care related to your symptoms since [enrollment date]? Select all that apply.
    - Yes - Doctor's Office or Urgent Care
    - Yes - Pharmacy (drugstore)
    - Yes - Emergency Department
    - Yes - Hospital (admitted)
    - Yes - A provider in Isolation & Quarantine
    - Yes - Via phone or telehealth visit
    - Yes - Other
    - None *🡪 If selected, skip to question 13*
11. How long ago was your most recent medical visit for your symptoms?
    - Less than seven days ago
    - 7-15 days ago
    - More than 15 days ago
12. The next questions are about your quality of life related to your health. We will ask you these questions two times-- first about your current health and quality of life, then about your health and quality of life prior to [enrollment date] when you were tested for COVID-19 through our study. First, please answer these questions about your current health.
13. Would you say that in general your health is:
    - Excellent
    - Very good
    - Good
    - Fair
    - Poor
    - Don’t know/ not sure
    - Refused
14. Now thinking about your physical health, which includes physical illness and injury, for how many days during the past 30 days was your physical health NOT good?
    - Number 1-30
    - None
    - Don’t know/Not sure
    - Refused
15. Now thinking about your mental health, which includes stress, depression, and problems with emotions, for how many days during the past 30 days was your mental health NOT good?
    - Number 1-30
    - None
    - Don’t know/Not sure
    - Refused
16. During the past 30 days, for about how many days did poor physical or mental health keep you from doing your usual activities such as self-care, work, or recreation?
    - Number 1-30
    - None
    - Don’t know/Not sure
    - Refused
17. These next questions are about physical, mental, or emotional problems or limitations you may have in your daily life. Are you LIMITED in any way in any activities because of any impairment or health problem?
    - Yes
    - No *🡪If selected, skip to question 23*
    - Don't know/Not sure *🡪If selected, skip to question 23*
    - Refused*🡪If selected, skip to question 23*
18. What is the MAJOR impairment or health problem that limits your activities? (*DO NOT READ OPTIONS. Code only one category that represents the major impairment.*)
    - Arthritis/rheumatism
    - Back or neck problem
    - Fractures, bone/joint injury
    - Walking problem
    - Lung/breathing problem
    - Hearing problem
    - Eye/vision problem
    - Heart problem
    - Stroke problem
    - Hypertension/high blood pressure
    - Diabetes
    - Cancer
    - Depression/anxiety/emotional problem
    - Other impairment/problem
    - Don't know/Not sure
    - Refused
19. For HOW LONG have your activities been limited because of your major impairment or health problem? (*Do Not Read Options. Code using respondent's unit of time.*)
    - Days
    - Weeks
    - Months
    - Years
    - Don’t know/Not sure
    - Refused
20. Because of any impairment or health problem, do you need the help of other persons with your PERSONAL CARE needs, such as eating, bathing, dressing, or getting around the house?
    - Yes
    - No
    - Don’t know/Not sure
    - Refused
21. Because of any impairment or health problem, do you need the help of other persons in handling your ROUTINE needs, such as everyday household chores, doing necessary business, shopping, or getting around for other purposes?
    - Yes
    - No
    - Don’t know/Not sure
    - Refused
22. We are now going to repeat the same health and quality of life questions. This time, please think and respond about your health prior to when you were tested for COVID-19 through our study on [enrollment date]. Would you say that in general your health (prior to [enrollment date]) was:
    - Excellent
    - Very good
    - Good
    - Fair
    - Poor
    - Don’t know/ not sure
    - Refused
23. Now thinking about your physical health, which includes physical illness and injury, for how many days during the 30 days prior to [enrollment date] was your physical health NOT good?
    - Number 1-30
    - None
    - Don’t know/Not sure
    - Refused
24. Now thinking about your mental health, which includes stress, depression, and problems with emotions, for how many days during the 30 days prior to [enrollment date] was your mental health NOT good?
    - Number 1-30
    - None
    - Don’t know/Not sure
    - Refused
25. During the 30 days prior to [enrollment date], for about how many days did poor physical or mental health keep you from doing your usual activities such as self-care, work, or recreation?
    - Number 1-30
    - None
    - Don’t know/Not sure
    - Refused
26. These next questions are about physical, mental, or emotional problems or limitations you may have in your daily life. Prior to [enrollment date], were you LIMITED in any way in any activities because of any impairment or health problem?
    - Yes
    - No*🡪If selected, skip to question 32*
    - Don't know/Not sure*🡪If selected, skip to question 32*
    - Refused*🡪If selected, skip to question 32*
27. What is the MAJOR impairment or health problem that limited your activities? (*DO NOT READ OPTIONS. Code only one category that represents the major impairment.*)
    - Arthritis/rheumatism
    - Back or neck problem
    - Fractures, bone/joint injury
    - Walking problem
    - Lung/breathing problem
    - Hearing problem
    - Eye/vision problem
    - Heart problem
    - Stroke problem
    - Hypertension/high blood pressure
    - Diabetes
    - Cancer
    - Depression/anxiety/emotional problem
    - Other impairment/problem
    - Don't know/Not sure
    - Refused
28. For HOW LONG had your activities been limited because of your major impairment or health problem? (*Do Not Read Options. Code using respondent's unit of time.*)
    - Days
    - Weeks
    - Months
    - Years
    - Don’t know/Not sure
    - Refused
29. Because of any impairment or health problem, did you need the help of other persons with your PERSONAL CARE needs, such as eating, bathing, dressing, or getting around the house?
    - Yes
    - No
    - Don’t know/Not sure
    - Refused
30. Because of any impairment or health problem, did you need the help of other persons in handling your ROUTINE needs, such as everyday household chores, doing necessary business, shopping, or getting around for other purposes?
    - Yes
    - No
    - Don’t know/Not sure
    - Refused
